# ﻿Another step through the crux: a new microendemic rock-dwelling *Paroedura* (Squamata, Gekkonidae) from south-central Madagascar

**DOI:** 10.3897/zookeys.1181.108134

**Published:** 2023-10-04

**Authors:** Costanza Piccoli, Francesco Belluardo, Javier Lobón-Rovira, Ivo Oliveira Alves, Malalatiana Rasoazanany, Franco Andreone, Gonçalo M. Rosa, Angelica Crottini

**Affiliations:** 1 CIBIO, Centro de Investigação em Biodiversidade e Recursos Genéticos, InBIO Laboratório Associado, Campus de Vairão, Universidade do Porto, 4485-661 Vairão, Portugal Museo Regionale di Scienze Naturali Turin Italy; 2 Departamento de Biologia, Faculdade de Ciências, Universidade do Porto, Rua do Campo Alegre s/n, 4169-007 Porto, Portugal Universidade do Porto Vairão Portugal; 3 BIOPOLIS Program in Genomics, Biodiversity and Land Planning, CIBIO, Campus de Vairão, 4485-661 Vairão, Portugal Universidade do Porto Porto Portugal; 4 MRSN, Museo Regionale di Scienze Naturali, Via G. Giolitti, 36, I-10123 Turin, Italy BIOPOLIS Program in Genomics, Biodiversity and Land Planning, CIBIO, Campus de Vairão Vairão Portugal; 5 Mention Zoologie et Biodiversité Animale, Université d’Antananarivo, Antananarivo, Madagascar Université d’Antananarivo Antananarivo Madagascar; 6 Institute of Zoology, Zoological Society of London, Regent’s Park, NW1 4RY London, UK Institute of Zoology, Zoological Society of London London United Kingdom; 7 Centre for Ecology, Evolution and Environmental Changes (cE3c) & Global Change and Sustainability Institute (CHANGE), Faculdade de Ciências da Universidade de Lisboa, Bloco C2, Campo Grande, 1749-016 Lisboa, Portugal Centre for Ecology, Evolution and Environmental Changes & Global Change and Sustainability Institute Lisboa Portugal

**Keywords:** Anja Reserve, dry-deciduous forests, Haute Matsiatra, integrative taxonomy, Tsaranoro Valley

## Abstract

Using an integrative taxonomic approach including genetic and morphological data, we formally describe a new microendemic gecko species belonging to the *Paroedurabastardi* clade, previously referred to as *P.bastardi* D. We name this taxon currently known from Anja Reserve and Tsaranoro Valley Forest (south-central Madagascar), as *P.manongavato***sp. nov.** The new species differs from other species of the *P.bastardi* clade by ≥ 12.4% uncorrected *p*-distance at the mitochondrial 16S rRNA gene and it forms a monophyletic group in the COI mtDNA phylogenetic tree. It lacks haplotype sharing at the nuclear KIAA1239 and CMOS genes with the other species of the same complex, including the syntopic *P.rennerae*. Given its limited extent of occurrence and high levels of habitat fragmentation linked to forest clearances and fires, we propose the IUCN Red List Category of Critically Endangered, based on the B1ab(iii) criterion. The conservation value of Anja Reserve and Tsaranoro Valley Forest is remarkable. Preserving the remaining deciduous forest habitat is of paramount importance to protect these narrow-range reptile species.

## ﻿Introduction

The fauna of Madagascar has evolved in relative isolation from ca. 88 million years ago, following the tectonic separation from Africa, Antarctica, Australia and lastly the India-Seychelles landmass (Ali and Aitchison 2008; Crottini et al. 2012a; Collins et al. 2022). The complete isolation of Madagascar preceded a period of drastic species decline and turnover resulting from the mass extinction of the K/T boundary (ca. 65 million years ago) (Crottini et al. 2012a; Antonelli et al. 2022; Ali and Hedges 2023). The geological history and topography of the island (Stephenson et al. 2021) shaped its climatic conditions, resulting today in a wide diversity of biomes, from the eastern humid rainforest to the western dry and southern sub-arid biomes, providing a complex setting for the evolution and diversification of its biota (Crottini et al. 2012a; Brown et al. 2014; Antonelli et al. 2022). Madagascar is renowned for its native reptile diversity (Glaw and Vences 2007; Crottini et al. 2012a; Nagy et al. 2012; Uetz et al. 2023), currently amounting to over 430 native nominal species, of which 98% are endemic to the island (Antonelli et al. 2022; Goodman 2022; Uetz et al. 2023). A third of reptile species are considered microendemic (i.e. with distributional range less than 1,000 km^2^; *sensu*Brown et al. (2014)) and their diversity peaks in both the north-eastern humid and south-western dry biomes (Brown et al. 2016; Antonelli et al. 2022).

Despite a rapid increase in species cataloguing over the last three decades, numerous candidate species still await a taxonomic assessment and formal description (Nagy et al. 2012; Antonelli et al. 2022). This is especially true for gekkonid lizards, one of the most speciose reptile groups in Madagascar, in which molecular evidence uncovered several deeply divergent mitochondrial lineages, currently considered candidate species (Nagy et al. 2012). Using an integrative taxonomic approach that combines independent lines of evidence (Padial et al. 2010), several taxa have already been described in the last decade (e.g. Crottini et al. 2011; Ratsoavina et al. 2011, 2020; Köhler et al. 2019), also as the result of species complexes revisions (e.g. Hawlitschek et al. 2018; Miralles et al. 2021; Vences et al. 2022).

The genus *Paroedura* Günther, 1879 currently contains 24 nominal species, including two species endemic to the Comoros (Uetz et al. 2023). They are widely distributed across Madagascar, with the largest species diversity found in the north-eastern rainforests and south-western dry regions (Glaw and Vences 2007; Jackman et al. 2008; Glaw et al. 2018; Miralles et al. 2021). *Paroedura* species are found in different climates and microhabitats, with species restricted to rainforest (e.g. *P.masobe* Nussbaum & Raxworthy, 1994) or montane (e.g. *P.ibityensis* Rösler & Krüger, 1998) habitats; and there are ground-dwellers (e.g. *P.picta* (Peters, 1854)), karst specialists (e.g. *P.spelaea* Glaw, Köhler & Vences, 2018) or granitic rock-dwellers (e.g. *P.rennerae* Miralles, Bruy, Crottini, Rakotoarison, Ratsoavina, Scherz, Schmidt, Köhler, Glaw & Vences, 2021). Recent studies (Glaw et al. 2018; Köhler et al. 2019; Miralles et al. 2021) contributed to the description of five species in the *P.oviceps* (Boettger, 1881) and *P.bastardi* (Mocquard, 1900) clades (*P.fasciata* Glaw, Köhler & Vences, 2018, *P.kloki* Glaw, Köhler & Vences, 2018, *P.spelaea*, *P.neglecta* Köhler, Vences, Scherz & Glaw, 2019 and *P.rennerae*), the resurrection of *P.guibeae* Dixon & Kroll, 1974 and the re-description of *P.bastardisensu stricto.* The monophyly of the *P.bastardi* clade has been recovered by several authors (e.g. Jackman et al. 2008; Glaw et al. 2018; Köhler et al. 2019). The taxonomic revision of this clade by Miralles et al. (2021) also resulted in the identification of a candidate species (*P.bastardi* D, ZCMV 12790) from Anja Reserve (Haute Matsiatra Region), later found also by Belluardo et al. (2021) in another nearby locality (referred in that contribution as P.sp. aff.bastardi Lineage D).

This study contributes to advancing the taxonomy and systematics of the *Paroedurabastardi* clade by providing: 1) a formal description of *P.bastardi* D, including our proposal of its conservation status; 2) new morphological, molecular and distributional information for *P.rennerae*; and 3) a new phylogenetic hypothesis for the diversification of this clade.

## ﻿Materials and methods

### ﻿Abbreviations of analysed materials

The abbreviations for field numbers are: **ACZCV**, field numbers of Angelica Crottini; **FAZC**, field numbers of Franco Andreone; **FGMV**, field numbers of Frank Glaw and Miguel Vences; **FGZC** or **F**, field numbers of Frank Glaw; **ZCMV** or **M**, field numbers of Miguel Vences; **GA**, field numbers of Gennaro Aprea; **MirZC**, field numbers of Aurélien Miralles; **PSG**, field numbers of Philip-Sebastian Gehring. **ACZC** and **ACP** are tissue and extraction codes of Angelica Crottini. The abbreviations for institutional collections are: **MRSN**, Museo Regionale di Scienze Naturali, Torino; **UADBA**, Département de Biologie Animale de Université d’Antananarivo; **PBZT**, Parc Botanique et Zoologique de Tsimbazaza; **ZMA**, Zoölogisch Museum Amsterdam; and **ZSM**, Zoologische Staatssammlung München.

### ﻿Field sampling

**Figure 1.**
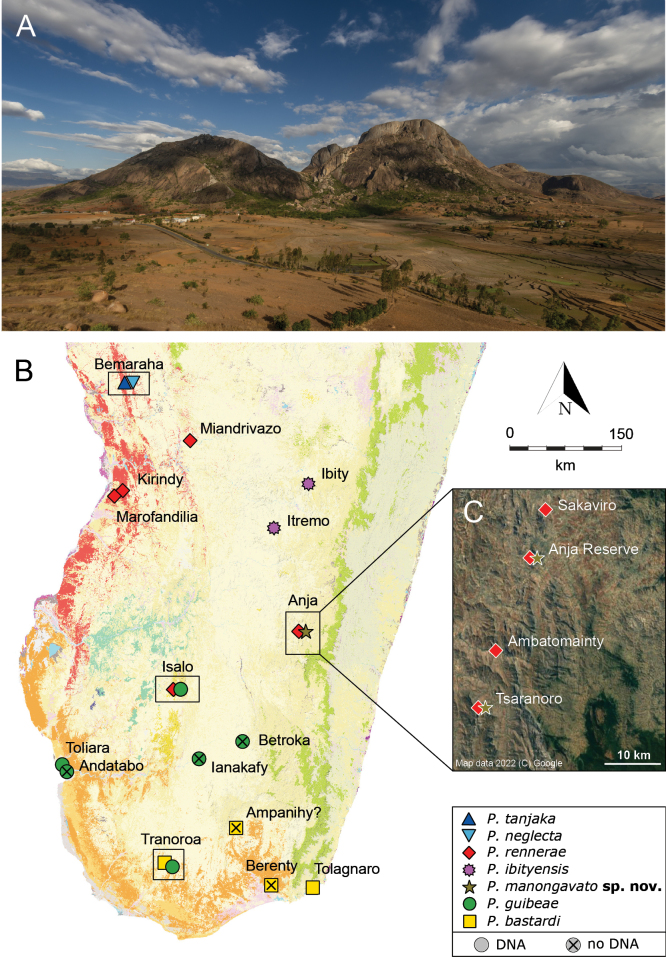
**A** Panoramic view of Anja Reserve, south of the city of Ambalavao (Fianarantsoa Province) **B** distribution of the different species within the *P.bastardi* clade, updated from Miralles et al. (2021) by adding Itremo for *P.ibityensis* (FGMV 2001-D37) and Ianakafy for *P.guibeae* (FMNH 73059). The exact geographic position of Ampanihy remains uncertain. Species symbol colours match the colours of the phylogenetic trees and haplotype networks. Black rectangles highlight localities with sympatric lineages. The map shows the remaining primary vegetation of Madagascar (https://kew.iro.bl.uk/): evergreen humid forest (green), deciduous dry forest (red), spiny arid forest (orange), tapia forest (yellow), western sub-humid forest (blue), mangroves (purple), cultivation (pink) and south western coastal bushland (grey) **C** satellite image with new records of *P.rennerae* and *P.manongavato* sp. nov. in the area south of Ambalavao. Map data 2022 (C) Google. Photographs by JLR.

Eighteen tissue samples and ten individuals of *Paroedurarennerae* and seven tissue samples and four individuals of *P.bastardi* D were collected during opportunistic surveys in the study area at dusk or at night during three visits in 2009, 2014 and 2018 (Fig. 1; Suppl. material 1). In 2009 and 2014, the surveys were focused on Anja Reserve, while in 2018, several forest fragments surrounding the Andringitra Massif were visited, including Anja Reserve and Tsaranoro (Belluardo et al. 2021). Surveys were conducted both in the forested and nearby areas. GPS coordinates and high-resolution photographs were taken for each individual encountered. All vouchers were fixed in 96% ethanol and then stored in 70% ethanol. Each voucher was labelled with a unique field code (ACZCV) and later deposited at ZSM or UADBA. The complete list of tissue samples and specimens analysed in this study is available in Suppl. material 1.

### ﻿Morphological analyses

**Figure 2.**
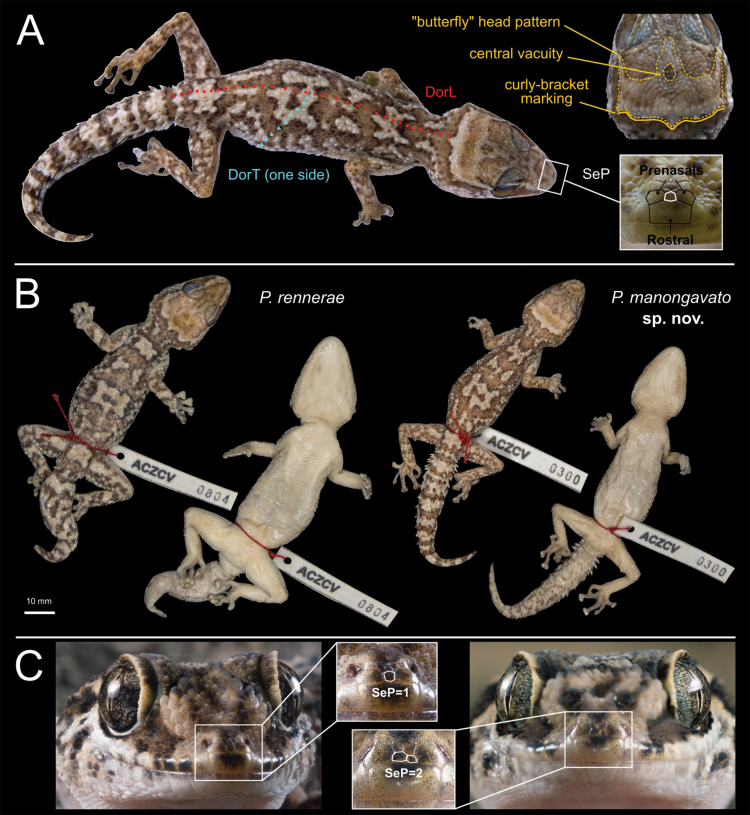
Morphological diagnostic characters and colourations of *Paroedurarennerae* and *P.manongavato* sp. nov. **A** morphological diagnostic characters defined and examined in this study (DorL, DorT and SeP, for definitions, see Methods section) and head pattern details (*sensu*Miralles et al. (2021)) of *P.manongavato* sp. nov. holotype **B** comparison of dorsal and ventral colouration of a preserved specimen of *Paroedurarennerae* (ZSM 8/2023, ACZCV 0804), after four years in 70% ethanol and preserved holotype of *P.manongavato* sp. nov. (ZSM 9/2023, ACZCV 0300), after nine years in 70% ethanol. Colours calibrated with ColorChecker **C** comparison of character SeP in *P.rennerae* (right) and *P.manongavato* sp. nov. (left). Photographs by CP and JLR. Scale bar: 10 mm (**B**).

We collected the same morphological quantitative and qualitative characters analysed in Miralles et al. (2021). Three individuals (ACZCV 0528, ACZCV 0777 and ACZC10441) of the new species were examined through photographic material using ImageJ (Abramoff et al. 2004) (Suppl. material 2). The following morphological measures (in mm) were taken by CP using a dial caliper Wiha dialMax to the nearest 0.1 mm: **SVL**, snout–vent length; **TL**, tail length; **HL**, head length from the anterior margin of the ear opening to the tip of the snout; **HW**, maximum head width; **HH**, maximum head height behind the eyes; **distE**, minimum distance between the bony edges of the orbits in dorsal view; **AGL**, axilla to groin distance; **ED**, maximum eye diameter; **EO**, maximal ear opening; **FoL**, foot length (from ankle to tip of the 3^th^ toe, left side); **HAL**, hand length, distance between the wrist and the tip of the longest finger (until the insertion of the claw, which is not included, left side); **TIBL**, tibia length (between the ankle and the knee flexed at 90°, left side). The following qualitative characters were inspected by CP and AC: **IO**, minimum number of interorbital scales at mid-eye level; **SO**, number of granular scales across the upper eyelid counted transversally; **SnoutS**, arrangement of the mediodorsal scale rows of the snout tip: mostly forming two transverse rows of granules in contact (c), separated by a third median row (s) or intermediate pattern (i); **DigC**, colouration of the toes, uniform (u) or bicolour (b). We assessed three additional morphological traits: **DorL**, number of dorsal-enlarged keeled scales counted along the median longitudinal row from the middle section of the nuchal curly-bracket to the base of the tail; **DorT**, number of dorsal-enlarged keeled scales counted transversally at mid-body from flank to flank and including the dorsal line; and **SeP**, number of scales of the snout tip in contact with rostral scale separating the prenasals (Fig. 2A). We also examined the dorsal pattern (*sensu*Miralles et al. (2021)), focusing on the different concentration of melanophores across the body in defining contrasting markings.

We performed multivariate statistical analysis in R 4.1.3 (R Core Team 2022), using the packages FactoMineR (version 2.8; Lê et al. (2008)) and missMDA (Josse and Husson 2016), which allowed us to analyse datasets containing both quantitative and qualitative variables. We integrated our newly obtained data for *Paroedurabastardi* D and *P.rennerae* to the morphological dataset used in Miralles et al. (2021), excluding adults with several missing data and juveniles. A Principal Component Analysis was conducted including 14 variables (one qualitative and 13 quantitative characters) from 29 adults and subadults. Morphological measurements were corrected by size (SVL), centred and the data scaled prior the components analysis. Finally, we used ggplot2 (Wickham 2016) for visualisation of the principal components and variables contribution to the variance.

### ﻿Molecular analyses

We extracted total genomic DNA using proteinase K digestion (20 mg/ml concentration), followed by a standard salt-extraction protocol (Bruford et al. 1992). Information on samples and sequences analysed in this study (comprising both newly-generated and previously-published sequences with respective GenBank accession numbers) are available in Suppl. material 1. We amplified the following markers: a fragment of ca. 550 bp of the 3’ terminus of the mitochondrial 16S rRNA gene (16S); a fragment of ca. 360 bp of the mitochondrial 12S rRNA gene (12S); a fragment of ca. 600 bp of the mitochondrial protein-coding gene cytochrome oxidase subunit 1 (COI); a fragment of ca. 580 bp of the mitochondrial protein-coding NADH dehydrogenase Subunit 2 (ND2); a fragment of ca. 870 bp of the nuclear-encoded leucine-rich repeat and WD repeat-containing protein 1239 (KIAA1239); a fragment of ca. 430 bp of the nuclear-encoded oocyte maturation factor (CMOS); a fragment of ca. 370 bp of the nuclear-encoded phosducin (PDC); two fragments of ca. 970 bp and 1,000 bp, respectively, of the nuclear-encoded sacsin (SACS). Information on molecular markers, primers and amplification conditions for each amplified marker are provided in Table 1. Standard polymerase chain reactions (PCR) were performed in a final volume of 25 μl, using the following reagents: 5 µl of 5× Green GoTaq Flexi Buffer Promega; 4 µl of MgCl_2_ Promega (25 mM), 1 µl of each of the primers (10 pmol/µl); 0.4 µl of dNTPs Promega (10 mM) and 0.1 µl of GoTaq Flexi DNA Polymerase Promega (5 U/µl). Both reactions of nested PCRs were performed in the final volume of 25 μl described above. In the second reaction of the nested PCRs, 1 μl of the first reaction's product was used. The purified PCR products were sequenced using Sanger sequencing on a 3730 x l sequencer at Macrogen Inc. (Madrid, Spain). Sequences were visually checked and manually edited, when necessary, in BioEdit (version 7.2.5; Hall 1999). Newly-generated sequences were deposited in GenBank (12S: OR471406; 16S: OR471407–OR471422; CMOS: OR472336–OR472360; KIAA1239: OR472361–OR472384; ND2: OR472385; PDC: OR472386; SACS: OR472387).

**Table 1.** Molecular markers, primers (forward and reverse) and amplification conditions for each marker. Amplification protocols marked with an asterisk are modified from the original publication.

Marker	Primers	Primer sequence (5’-3’)	Reference	Amplification conditions
12S rRNA	12SA-L	AAACTGGGATTAGATACCCCACTAT	Kocher et al. (1989)	94 °C (90 s), [94 °C (45 s), 52 °C (45 s), 72 °C (90 s)×33], 72 °C (300 s)*
	12SB-H	GAGGGTGACGGGGCGGTGTGT	Palumbi et al. (1991)	
16S rRNA	16sar-L	CGCCTGTTTATCAAAAACAT	Palumbi et al. (1991)	94 °C(90 s), [(94 °C (45 s), 55 °C (45 s), 72 °C (90 s)×33], 72 °C (600 s)
	16sbr-H	CCGGTCTGAACTCAGATCACGT		
COI	RepCOI-F	TNTTMTCAACNAACCACAAAGA	Nagy et al. (2012)	94 °C (180 s), [94 °C (40 s), 48.5 °C (30 s), 72 °C (60 s)×40], 72 °C (420 s)
	RepCOI-R	ACTTCTGGRTGKCCAAARAATCA		
ND2	ND2 F17	TGACAAAAAATTGCNCC	Macey et al. (1997)	94 °C (90 s), [94 °C (45 s), 47 °C (45 s), 72 °C (90 s)×34], 72 °C (600 s)*
	ALAR2	AAAATRTCTGRGTTGCATTCAG		
KIAA 1239 nested PCR	KIAA1239F1	CARCCTTGGGTNTTYCARTGYAA	Shen et al. (2011)	94 °C (240 s), [94 °C (45 s), 45 °C (40 s), 72 °C (120 s)×45], 72 °C (600 s)
	KIAA1239R1	ACMACAAAYTGGTCRTTRTGNGT	Shen et al. (2012)	
	KIAA1239NF1	GAGCCNGAYATHTTYTTYGTNAA		94 °C (240 s), [94 °C (45 s), 45 °C (40 s), 72 °C (120 s)×35], 72 °C (600 s)
	KIAA1239NR1	TTCACRAANCCMCCNGAAAAYTC		
CMOS	CO8	GCTTGGTGTTCAATAGACTGG	Han et al. (2004)	94 °C (180 s), [94 °C (45 s), 51 °C (45 s), 72 °C (60 s)×36], 72 °C (360 s)
	CO9	TTTGGGAGCATCCAAAGTCTC		
PCD	PHOF2	AGATGAGCATGCAGGAGTATGA	Bauer et al. (2007)	95 °C (120 s), [95 °C (35 s), 50.4 °C (35 s), 72 °C (95 s)×35], 72 °C (240 s)*
	PHOR1	TCCACATCCACAGCAAAAAACTCCT		
SACS frag. 1 nested PCR	SACSF1	AARGARATHTGGAARACNGAYAC	Shen et al. (2012)	94 °C (240 s), [94 °C (45 s), 45 °C (40 s), 72 °C (120 s)×45], 72 °C (600 s)
	SACSR1	GCYTTNGCRTCRTCNGCRTTYTG		
	SACSNF1	CAYCCYGAAGGAMGNGTNGCNAA		94 °C (240 s), [94 °C (45 s), 45 °C (40 s), 72 °C (120 s)×35], 72 °C (600 s)
	SACSNR1	GCWACYTCYCKNGGDATRTC		
SACS frag. 2 nested PCR	SACSF2	AAYATHACNAAYGCNTGYTAYAA	Shen et al. (2012)	94 °C (240 s), [94 °C (45 s), 45 °C (40 s), 72 °C (120 s)×45], 72 °C (600 s)
	SACSR2	GCRAARTGNCCRTTNACRTGRAA		
	SACSNF2	TGYTAYAAYGAYTGYCCNTGGAT		94 °C (240 s), [94 °C (45 s), 45 °C (40 s), 72 °C (120 s)×35], 72 °C (600 s)
	SACSNR2	CKGTGRGGYTTYTTRTARTTRTG		

We conducted molecular analyses, based on five independent datasets with different purposes:

- **Dataset 1** contains 41 16S sequences belonging to all nominal and candidate species of the *Paroedurabastardi* clade and comprising both newly-generated and GenBank available sequences (Suppl. material 1). Mean and minimum and maximum values of uncorrected pairwise genetic distances transformed into percentage were calculated within and between groups (intraspecific and interspecific *p*-distances, respectively) using TaxI2 from iTaxoTools (Vences et al. 2021).
- **Dataset 2** contains 89 COI sequences (all sequences already available in GenBank; Suppl. material 1) for all nominal and candidate species of the *Paroedurabastardi* clade. A phylogenetic hypothesis was inferred for this marker under the Maximum Likelihood (ML) optimality criterion in MEGA X (Kumar et al. 2018), using the Kimura 2-parameter substitution model, NNI branch swapping and 500 bootstrap replicates to assess the robustness of the nodes. The scope of the analysis was to assess the monophyly of the samples belonging to *P.bastardi* D from Anja and Tsaranoro.
- **Datasets 3 and 4** contain the alignments of phased nuclear loci for KIAA1239 and CMOS, including both newly-generated and GenBank sequences for all nominal and candidate species of the *Paroedurabastardi* clade. Three shorter (less than 810 bp) sequences (GenBank Accession numbers: OR472364, OR472365 and OR472381) were removed from *Dataset 3*. All the remaining KIAA1239 sequences (75 sequences) were trimmed to equal length (869 bp). **Dataset 4** contains 73 sequences of equal length (434 bp). PHASE algorithm (Stephens et al. 2001) implemented in DnaSP (version 6.12.03; Rozas et al. (2017)) was used to determine the most probable inferred haplotype for each nuclear sequence, setting 500 iterations, one thinning interval, 100 burn-in iterations and a posterior threshold of 0.9 (Suppl. material 4). For each marker, phased alignments were used to build a Maximum Likelihood tree using the Jukes-Cantor substitution model in MEGA X, that was inputted in HaploViewer software (http://www.cibiv.at/~greg/haploviewer) to infer the haplotype network following the approach proposed by Salzburger et al. (2011). For visualisation purposes and following the approach used in Miralles et al. (2021), despite the occurrence of more than one mitochondrial lineage in both *P.tanjaka* and *P.guibeae*, we consider this as intraspecific diversity. These datasets were compiled to evaluate the amount of allele sharing between populations of the different taxa of the *P.bastardi* clade. Lack of haplotype sharing at the phased KIAA1239 and CMOS genes amongst individuals belonging to different mitochondrial lineages and which occur in sympatry, was considered as further evidence of independent evolution.
- **Dataset 5** (a slightly modified version of the dataset assembled by Miralles et al. (2021) to assess the interspecific phylogenetic relationships within the *P.bastardi* clade) contains sequences for a representative for each nominal and candidate species of the *Paroedurabastardi* clade for nine nuclear markers (ACM4, CMOS, KIAA1239, MXRA5, PDC, PRLR, RAG1, SACS and TTN) and five mitochondrial markers (12S, 16S, ND2, ND4 and COI), using *P.picta* as outgroup. Sequences of eight molecular markers *P.bastardi* D were included to the dataset published in Miralles et al. (2021). The input file for phylogenetic analysis was prepared using PipeLogeny in R 4.1.3 (Muñoz-Pajares et al. 2019; R Core Team 2022). Through this pipeline, sequences were aligned with MAFFT (version 7.490; Katoh and Standley (2013)) and the best-fit partitioning scheme and models of molecular evolution for the phylogenetic analysis were identified with PartitionFinder (version 2.1.1; Lanfear et al. (2017); Suppl. material 3). Bayesian Inference was conducted using MrBayes (version 3.2.6; Ronquist et al. (2012)) on the CIPRES Science Gateway (Miller et al. 2010). Two runs of 10 million generations each were performed, setting four independent Markov chains (with default heating values) for each run, sampling every 1,000 generations and setting the burn-in to 25%. Average standard deviation of split frequencies was examined and log likelihood values associated with the posterior probability distribution were plotted in Tracer 1.7.2 (Rambaut et al. 2018) to infer the stationarity of the chains and assess their reciprocal convergence. The 50% majority-rule consensus tree was visualised in FigTree (version 1.4.4; Rambaut (2018)). The analysis aimed at inferring the phylogenetic relationships of *P.bastardi* D within the *P.bastardi* clade.

### ﻿Evaluation of conservation status

We computed the Area of Occupancy (AOO) using a grid cell width of 2 km and the Extent of Occurrence (EOO) in GeoCAT (Bachman et al. 2011) of *Paroedurabastardi* D. In this analysis, we considered the distributional records provided in Suppl. material 1. Following the IUCN Red List guidelines (IUCN Standards and Petitions Committee 2022), we propose an evaluation of the conservation status of this new taxon.

## ﻿Results

### ﻿Morphological analyses

**Figure 3.**
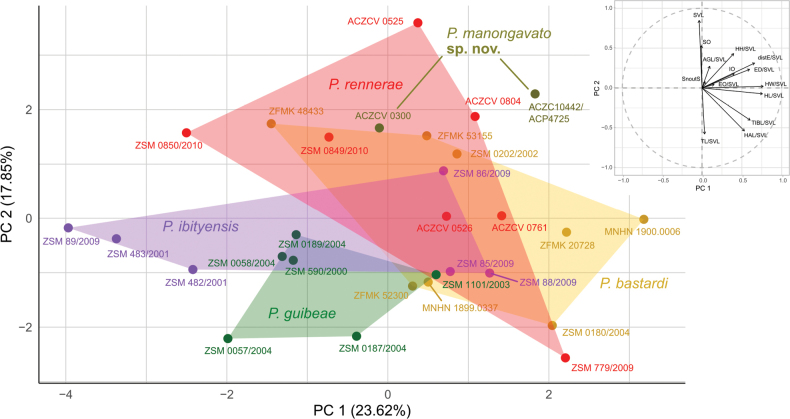
Scatterplot of Principal Component Analysis (axes: PC1 and PC2) showing the morphological variation in five lineages of the *Paroedurabastardi* clade (adults and subadults). See Suppl. material 5 for further details. Lineages colours follow Miralles et al. (2021) and match the map, ML COI tree, haplotype networks and multilocus Bayesian phylogeny.

Morphological measurements and the assessment of the analysed morphological traits are available in Table 2. The morphological dataset and the contribution of each variable to the first five principal components (PCs) are shown in Suppl. material 5. In the first two PCs, all species of the *P.bastardi* clade included in the analysis show a high overlap between them without significant difference (Fig. 3). The highest contribution (> 0.7) to the variation in the first two PCs is represented by SVL-corrected head size (HL/SVL and HW/ SVL in PC1) and body size (SVL in PC2), as in Miralles et al. (2021).

**Table 2.** Morphological traits examined in this study. Individuals in light grey shaded cells were inspected through photographic material (Suppl. material 2). Detached tail marked with an asterisk. For abbreviations and definitions, see Methods section. Undet.: sex undetermined; NA: not available.

Species	*Paroedurarennerae*					*Paroeduramanongavato* sp. nov.			
Catalogue code	ZSM 7/2023 (ACZCV 0761)	ZSM 5/2023 (ACZCV 0526)	ZSM 6/2023 (ACZCV 0745)	ZSM 8/2023 (ACZCV 0804)	ZSM 4/2023 (ACZCV 0525)	ZSM 9/2023 (ACZCV 0300)	UADBA R-uncatalogued (ACZCV 0528)	UADBA R-uncatalogued (ACZCV 0777)	ACZC10441
Status	-	-	-	-	-	Holotype	Paratype	Paratype	-
Stage	Subadult	Subadult	Juvenile	Adult	Adult	Adult	Adult	Juvenile	Adult
Sex	Undet.	Female	Undet.	Male	Male	Female	Undet.	Undet.	Male
IO	5	3	5	5	5	6	6	6	6
SO (right/left)	5/5	5/6	5/5	4/4	5/5	5/4	4/5	4/4	4/4
SnoutS	c/i	i	i	s/i	c/i	s	s/i	s	s/i
DigC	u	u	u	u	u	u	u	u	u
DorL	30	30–31	29	29–30	31	33	30–32	29–30	31–34
DorT	17	15	17	16	16	19	NA	NA	NA
SeP	1	1	1	1	1	1	2	2	2
SVL	61.2	60.9	45.6	75.2	79.7	68.3	73	NA	NA
TL	40.0*	44.4	28.3	35.9	37.5	49.0	40	NA	NA
HL	18.5	18.9	14.3	22.3	22.4	19.9	24.1	NA	NA
HW	14.8	15.1	11.2	18.7	19.1	15.3	19.3	NA	NA
HH	7.8	7.9	5.4	9.3	18.6	8.7	NA	NA	NA
distE	2.9	2.7	2.1	3.8	3.2	3.1	3.3	NA	NA
AGL	26.2	23.2	20.6	33.0	32.8	30.9	NA	NA	NA
ED	4.5	5.0	4.2	6.0	5.9	5.6	5.4	NA	NA
EO	2.8	2.9	2.1	3.2	3.8	2.8	NA	NA	NA
HAL	7.4	7.4	5.7	8.4	8.8	7.1	7.5	NA	NA
FoL	8.7	7.6	5.6	10.0	10.3	9.3	8.9	NA	NA
TIBL	12.2	11.0	8.0	13.6	14.2	11.8	NA	NA	NA

### ﻿Molecular analyses

#### ﻿Dataset 1

It was possible to compute the mean and the minimum and maximum values of intraspecific uncorrected *p*-distance only for *Paroedurabastardi* D, *P.rennerae* and *P.guibeae*, as at least two samples were available for these taxa. This was 0.5% (0.0–1.0%), 0.9% (0.0–3.2%) and 8.2% (0.0–14.8%), respectively (Table 3). The interspecific distance between *P.bastardi* D and the six currently-described species of the *P.bastardi* clade ranged between 12.4% (minimum calculated distance between *P.bastardi* D and *P.guibeae*) and 17.8% (maximum calculated distance between *P.bastardi* D and *P.neglecta*) (Table 3). Amongst species of this clade, the smallest interspecific genetic distance is observed between *P.neglecta* and *P.tanjaka* (12.3%) and the highest value between *P.rennerae* and *P.neglecta* (19.2%) (Table 3).

**Table 3.** Percentage of mean and minimum and maximum uncorrected *p*-distance values (minimum and maximum values provided in brackets) in the mitochondrial fragment 16S for the *Paroedurabastardi* clade, where intraspecific values (in bold) are along the diagonal and intraspecific values are below the diagonal. n/c: not calculated.

	*P.manongavato* sp. nov.	*P.rennerae*	*P.bastardi*	*P.guibeae*	*P.ibityensis*	*P.neglecta*	*P.tanjaka*
***P.manongavato* sp. nov.**	**0.5**						
	**(0.0**–**1.0)**						
***P.rennerae***	14.8	**0.9**					
	(13.8–16.2)	**(0.0**–**3.2)**					
***P.bastardi***	16.5	18.0	**n/c**				
	(16.4–16.8)	(17.9–18.6)					
***P.guibeae***	14.1	15.2	16.8	**8.2**			
	(12.4–16.5)	(13.9–17.8)	(15.4–18.8)	**(0.0**–**14.8)**			
***P.ibityensis***	15.2	15.0	15.4	14.6	**n/c**		
	(14.7–15.4)	(14.4–15.3)	(15.4–15.4)	(13.7–16.1)			
***P.neglecta***	17.6	18.4	14.7	16.4	13.4	**n/c**	
	(16.5–17.8)	(18.3–19.2)	(14.7–14.7)	(15.5–18.9)	(13.4–13.4)		
***P.tanjaka***	14.3	14.3	16.4	13.6	12.5	12.3	**n/c**
	(13.5–14.6)	(14.2–15.3)	(16.4–16.4)	(12.6–15.7)	(12.5–12.5)	(12.3–12.3)	

#### ﻿Dataset 2

**Figure 4.**
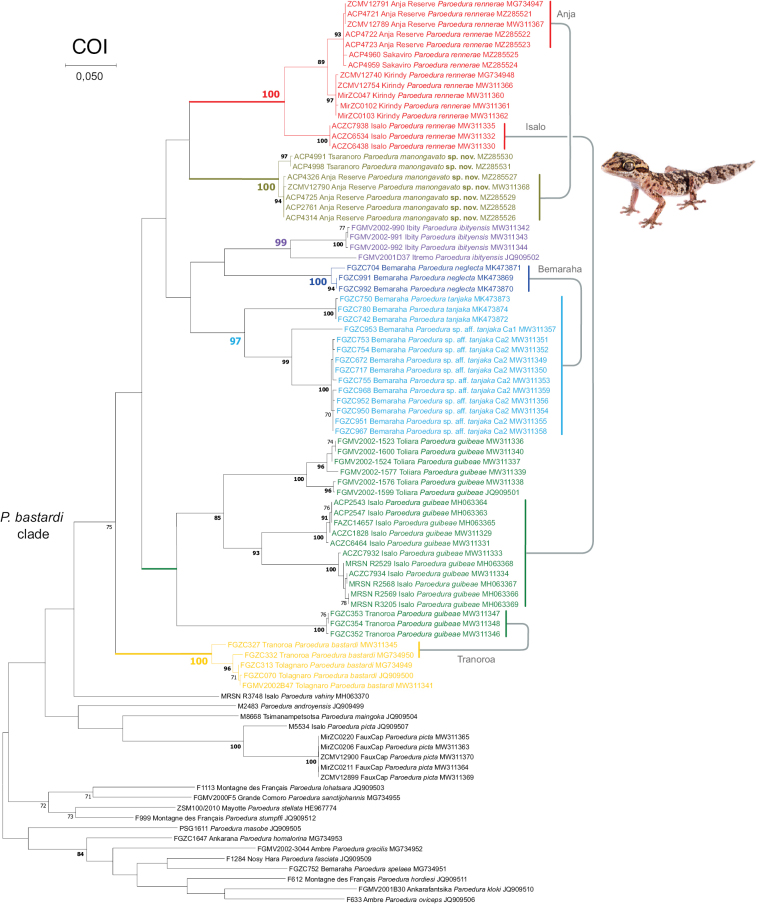
Maximum Likelihood phylogenetic tree of the *Paroedurabastardi* clade inferred with the COI alignment. Bootstrap values above 80 in bold, below 70 removed. Lineages colours follow Miralles et al. (2021) and match the map, scatterplot of the PCA, haplotype networks and multilocus Bayesian phylogeny. Photograph of *P.manongavato* sp. nov. by JLR.

In the mitochondrial ML tree (Fig. 4), all samples of *Paroedurabastardi* D formed a monophyletic clade which received full support (100% bootstrap support). Within this clade, samples group by locality. The new samples of *P.rennerae* analysed in this study cluster together with the samples of *P.rennerae* previously analysed by Miralles et al. (2021), forming a clade that received full support (100% bootstrap support). Additionally, in this case, samples group per locality. The sister relationship between *P.rennerae* and *P.bastardi* D did not receive statistical support.

#### ﻿Datasets 3 and 4

**Figure 5.**
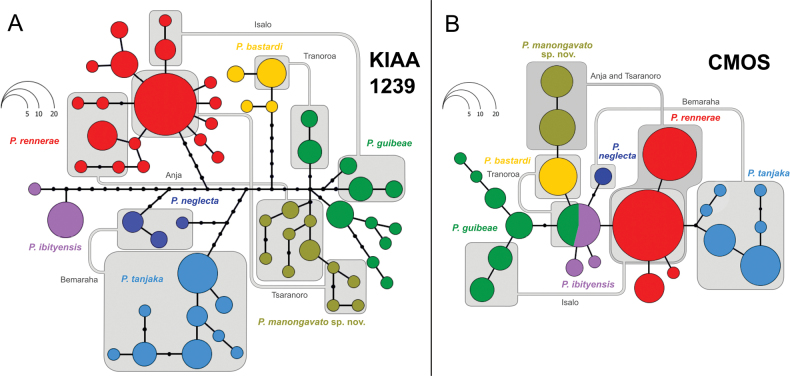
Haplotype networks inferred from the phased sequences of nuclear genes **A** KIAA1239 and **B**CMOS. Circles represent haplotypes, whose size is proportional to their frequency. Small black circles correspond to unsampled or extinct haplotypes and bars represent mutational steps between haplotypes. Colours match the different lineages of the *P.bastardi* clade. Haplotypes co-occurring in Anja, Tsaranoro, Isalo, Tranoroa and Bemaraha are connected with light grey lines and boxes.

Both haplotype networks (Fig. 5) were consistent with the results obtained in the ML COI tree (Fig. 4) and, amongst the two nuclear genes, the KIAA1239 alignment was found to be more variable (58 haplotypes). In terms of grouping, in both KIAA1239 and CMOS networks, the new samples of *Paroedurarennerae* and of *P.bastardi* D clustered together with the haplotypes of the same mitochondrial lineage. In terms of mutational steps, in the KIAA1239 network, the haplotypes of *P.guibeae* were the closest (minimum four steps), whereas in the CMOS network, only one mutational step separated *P.bastardi* D from *P.bastardi*. When considering both networks, haplotype sharing was only found in the CMOS network between *P.guibeae* and *P.ibityensis*.

#### ﻿Dataset 5

**Figure 6.**
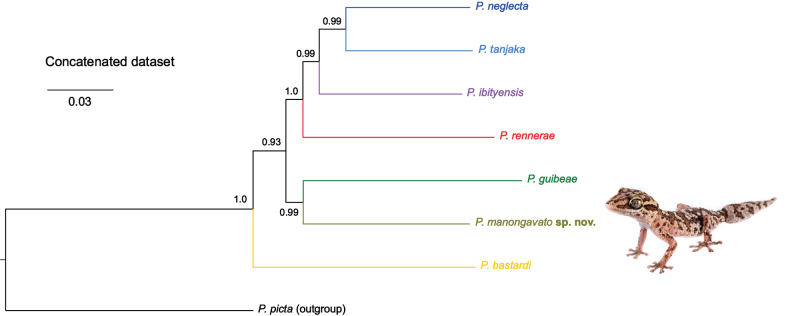
Multilocus Bayesian phylogeny of the *Paroedurabastardi* clade including the new species *P.manongavato* sp. nov. Colours match the lineages of map, scatterplot of the PCA, ML COI tree and haplotype networks. Photograph of *P.manongavato* sp. nov. by JLR.

The multilocus matrix used in the Bayesian Inference analyses included 10,468 sites. The best-fit partitioning scheme included 19 subsets (Suppl. material 3). We considered specific values of Posterior Probability (PP) when interpreting the phylogenetic relationships as strongly (≥ 0.99), moderately (0.95–0.98) and weakly (0.90–0.94) supported (Fig. 6). *Paroedurabastardi* was recovered in sister position to all the other lineages of the *P.bastardi* clade (PP = 1.0). *Paroeduraneglecta*, *P.tanjaka*, *P.ibityensis*, *P.rennerae*, *P.guibeae* and *P.bastardi* D form a weakly-supported clade (0.93). Within this clade, the sister relationship of *P.bastardi* D with *P.guibeae* was recovered with strong support (0.99). This group was found sister to a strongly-supported clade (1.0) containing *P.neglecta*, *P.tanjaka*, *P.ibityensis* and *P.rennerae*, where *P.rennerae* is sister to *P.neglecta*, *P.tanjaka* and *P.ibityensis*. *Paroeduraibityensis* is sister to *P.neglecta* and *P.tanjaka* (0.99) and the sister relationship of *P.neglecta* and *P.tanjaka* is strongly supported (0.99).

### ﻿Justification for species delimitation

We used the integration by congruence approach (Padial et al. 2010) and considered *Paroedurabastardi* D an independent evolutionary lineage, based on the identification of several independent lines of evidence to support its distinction.

Within the *P.bastardi* clade, *P.bastardi* D forms a strongly supported mitochondrial lineage (Fig. 4). In the multilocus phylogenetic analysis, this lineage is found to be sister to *P.guibeae* (Fig. 6). *Paroedurabastardi* D shows an interspecific uncorrected *p*-distance (16S) greater than 12.4% with the other currently-described species of the *P.bastardi* clade, which is greater than the lowest interspecific distance between the nominal species of the group (Table 3). *Paroedurabastardi* D does not share haplotypes with any other species of this clade in both nuclear genes analysed in this study (KIAA1239 and CMOS; Fig. 5), despite occurring in sympatry with *P.rennerae* in two localities, i.e. Anja Reserve and Tsaranoro Valley Forest (Figs 1, 4, 5). Regarding the morphology, although head size and SVL mostly contribute to the variation in the PCA, these characters are not sufficient alone in distinguishing the species as shown by the presence of overlap between *P.bastardi* D and *P.rennerae* in Fig. 3. As in Miralles et al. (2021), we inspected the external morphology of a limited number of traits known to be informative within this genus: (1) dorsal scales, (2) dorsal crossbands in juveniles, (3) spines on the original tail, (4) snout tip scales arrangement, (5) curly-bracket-shaped marking in the occipital region, (6) SnoutS, (7) SeP, (8) IO, (9) DorL, (10) DorT, (11) dorsal colouration in adults, (12) DigC, (13) anterior edge in the “butterfly” pattern on the head in both juveniles and adults, (14) central vacuity in the “butterfly” pattern on the head in both juveniles and adults and (15) SVL. In addition to these, we also inspected the concentration of melanophores across the body. The concordance between mitochondrial (i.e. COI) and nuclear (i.e. KIAA1239 and CMOS) markers, the molecular evidence for reproductive isolation between sympatric populations of *P.bastardi* D and *P.rennerae* in Anja Reserve and Tsaranoro Valley Forest and the identification of a combination of diagnostic morphological characters, support the distinction of *P.bastardi* D which is here formally described.

#### ﻿ Paroedura manongavato sp. nov.

Taxon classificationAnimaliaSquamataGekkonidae

1460CDFC-B8C5-5F79-B386-F44D6BDE07EC

https://zoobank.org/7B8D56F4-9E4F-4992-8112-51E599445AA4

**Figure 7.**
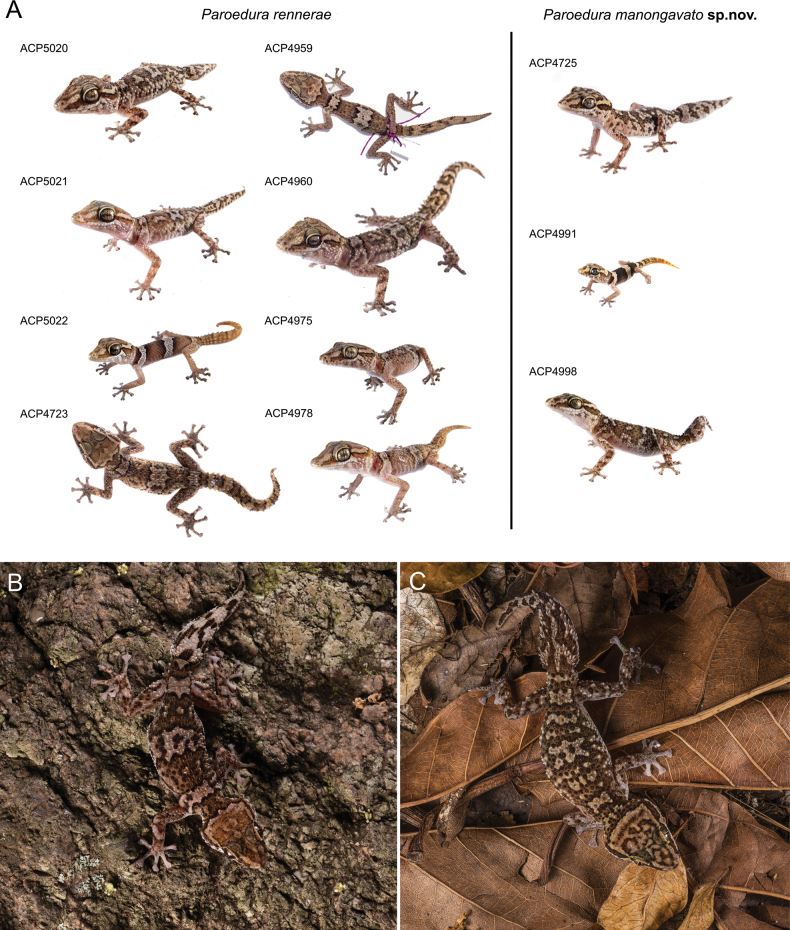
Dorsal or dorsolateral views, and colourations in life of juveniles and adults of *Paroedurarennerae* and *P.manongavato* sp. nov. **A** dorsal or dorsolateral view of *P.rennerae* from Ambatomainty (male, ACP5020; female, ACP5021; juvenile, ACP5022), from Anja (female, ACP4723), from Sakaviro (juvenile, ACP4959; female, ACP4960) and from Tsaranoro (ACP4975, ACP4978, both undetermined); dorsolateral view of *P.manongavato* sp. nov. from Anja (undetermined, ACP4725; juvenile, ACP4991) and from Tsaranoro (female, ACP4998) **B** dorsal colouration of *P.rennerae* (male, ACP4722) from Anja Reserve **C** dorsal colouration of *P.manongavato* sp. nov. (male, ACP5940) from Anja Reserve. Note the overall less spiky aspect of *P.manongavato* sp. nov. and the less contrasted ochre markings on dorsum, representing the two diagnostic characters that more likely help to discriminate between these two species when occurring in sympatry. Photographs by JLR.

**Figure 8.**
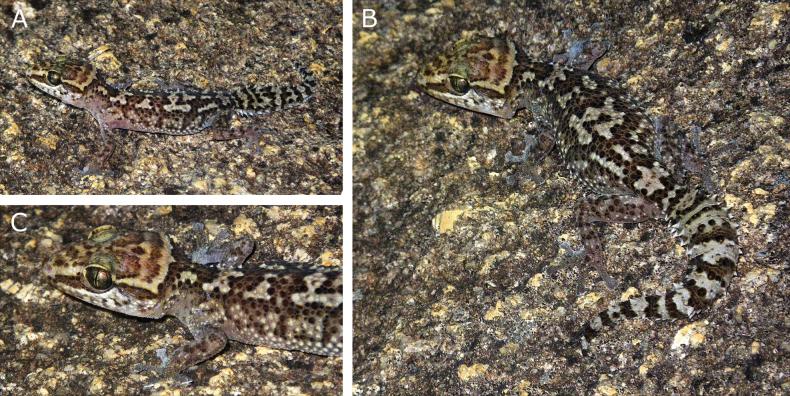
Living holotype of *Paroeduramanongavato* sp. nov. (ZSM 9/2023, ACZCV 0300), from Anja Reserve, photographed at night on the reserve boulders **A** dorsolateral view of the individual **B** dorsal view of the individual with detail on the original tail **C** detail of the head. Photographs by AC.

Figs 2 , 4 , 6 , 7A, C , 8

##### Remarks.

This species was previously referred to as *Paroedurabastardi* D by Miralles et al. (2021) and Paroedurasp. aff.bastardi Lineage D UCS by Belluardo et al. (2021). A single individual (ZCMV 12790) collected at Anja Reserve was molecularly examined in Miralles et al. (2021). Further samples have been analysed in Belluardo et al. (2021), which contributed to extend the species distribution to Tsaranoro Valley (ca. 25 km south of Anja Reserve).

##### Type locality.

Anja Reserve, 21.85098°S, 46.84270°E, elevation ca. 950 m a.s.l., Ambalavao, Fianarantsoa District, Haute Matsiatra Region, Madagascar.

##### Type material.

***Holotype*.**ZSM 9/2023 (ACZCV 0300; ACZC6935; ACP2761), adult female from Anja Reserve, 21.85098°S, 46.84270°E, elevation ca. 950 m a.s.l., Ambalavao, Fianarantsoa District, Haute Matsiatra Region, Madagascar, collected on the 27 November 2014 by Franco Andreone, Angelica Crottini and Gonçalo M. Rosa.

***Paratypes*.**UADBA R-uncatalogued (ACZCV 0528; ACZC10442; ACP4725), undetermined individual collected at Anja Reserve, 21.85223°S, 46.84404°E, elevation ca. 970 m a.s.l., on the 15 November 2018, by Francesco Belluardo, Javier Lobón-Rovira and Gonçalo M. Rosa; UADBA R-uncatalogued (ACZCV 0777; ACZC10941; ACP4991), juvenile individual collected at Tsaranoro Valley, Forêt Sacrée, 22.08491°S, 46.77545°E, elevation ca. 945 m a.s.l., on the 16 December 2018, by Francesco Belluardo, Javier Lobón-Rovira and Malalatiana Rasoazanany; and UADBA R-uncatalogued (ACZCV 0782; ACZC10953; ACP4998), female individual collected in Tsaranoro Valley, Forêt Sacrée, 22.08530°S, 46.77589°E, elevation ca. 955 m a.s.l., on the 16 December 2018, by Francesco Belluardo, Javier Lobón-Rovira and Malalatiana Rasoazanany.

##### Diagnosis.

*Paroeduramanongavato* sp. nov. can be distinguished from the other species in the *Paroedura* genus by the presence of three broad light crossbands on the dorsum in juveniles (the first one between forelimbs, the second one at mid-body and the third one between hind limbs) versus four light crossbands in all other species, with exception of the members of the *P.bastardi* clade and *P.oviceps*. Juvenile colouration in *P.vahiny* is not known. It can be distinguished from *P.gracilis* by absence (versus presence) of a white tip on the original tail, absence (versus presence) of a raised vertebral ridge on the dorsum and shorter forelimbs, which do not extend forward beyond tip of snout (versus exceeding the snout); from *P.masobe* by smaller (versus distinctively large) eyes and absence (versus presence) of a dorsal row of paired spines on the original tail; from *P.fasciata*, *P.homalorhina*, *P.hordiesi*, *P.vahiny* and *P.spelaea* by the presence of spines on the original tail (versus absence); from *P.gracilis*, *P.homalorhina*, *P.kloki*, *P.maingoka*, *P.masobe*, *P.oviceps* (from its type locality Nosy Be), *P.picta*, *P.spelaea*, most *P.tanjaka* individuals and *P.vahiny* by the presence of prominent dorsal tubercles arranged in regular longitudinal rows (versus rather irregular rows of dorsal tubercles).

Within the *Paroedurabastardi* clade, *P.manongavato* sp. nov. is characterised by the unique combination of the following characters: (1) presence of prominent dorsal-enlarged keeled scales arranged in regular longitudinal rows, (2) presence of three broad light crossbands on the dorsum in juveniles (unknown in subadults), (3) presence of spines on the original tail, (4) nostrils separated from rostral scale by prenasals, (5) presence of a curly-bracket-shaped marking in the occipital region, (6) mediodorsal scale rows of the snout tip forming two rows of enlarged scales separated by a third median row (SnoutS, state “s”, *sensu*Miralles et al. (2021)), (7) prenasal scales separated by two scales in contact with rostral, (8) minimum of six interorbital scales separating the eyes, (9) 30–34 dorsal enlarged keeled scales from the middle tip of the nuchal curly-bracket to the base of the tail in adults, (10) minimum of 19 transversal dorsal-enlarged keeled scales at mid-body in adults, (11) body dorsally brown with ochre patches organised into crossbands bordered by a thin dark brown line in adults, (12) uniform colouration of toes, (13) absence of concave anterior edge in the “butterfly” pattern (*sensu*Miralles et al. (2021)) on the head in both juveniles and adults, (14) presence of central vacuity in the light patch on the head in both juveniles and adults and (15) body size (SVL = 68.3–73 mm).

*Paroeduramanongavato* sp. nov. differs from *P.neglecta* and *P.tanjaka* by having the nostrils separated from the rostral scale by prenasals (versus nostrils in contact with the rostral); from *P.guibeae* by the presence of a curly-bracket-shaped marking in the occipital region (versus absence), by uniform colouration of toes (versus striped/bicolour toes), by mediodorsal scale rows of the snout tip forming two rows of enlarged scales separated by a third median row (versus mostly forming two rows of scales in contact; SnoutS, state “c”, *sensu*Miralles et al. (2021)) and by a larger adult body size (SVL > 68.3 mm versus SVL < 60 mm); from *P.ibityensis* by a larger adult body size (SVL > 68.3 mm versus SVL < 61 mm); from *P.bastardi* by the presence of a curly-bracket-shaped marking in the occipital region (versus absence) and by the absence of concave anterior edge in the “butterfly” pattern on the head in both juveniles and adults (versus presence only in juveniles).

*Paroeduramanongavato* sp. nov. differs from *P.rennerae*, with which it is found in sympatry in Anja and Tsaranoro, by having the mediodorsal scale rows of the snout tip forming two rows of enlarged granules separated by a third median row (versus mostly forming two rows of scales in contact), by having prenasal scales separated by two scales (versus one scale), by a minimum of six interorbital scales separating the eyes (versus a maximum of five interorbital scales separating the eyes), by 30–34 dorsal-enlarged keeled scales from the middle tip of the nuchal curly-bracket to the base of the tail in adults (versus 29–31), by a minimum of 19 transversal dorsal-enlarged keeled scales at mid-body in adults (versus 15–17), by a body dorsally brown with ochre patches organised into crossbands bordered by a thin dark brown line in adults (versus a thick blackish line), by presence of a central vacuity in the “butterfly” pattern on the head in juveniles and adults (versus absence) and by having a smaller body size (SVL 68.3–73 mm versus SVL 73.6–80.9 mm). Despite an overall less spiky appearance of the dorsal body, *Paroeduramanongavato* sp. nov. has a distinctly spikier regenerated tail than *P.rennerae* (Fig. 7). This trait, together with the dorsal colouration, seems to be the easiest way to differentiate these two species, when found in sympatry.

##### Description of the holotype.

Adult female (SVL = 68.3 mm) in good condition (Fig. 2B), with two ventral incisions.

Head triangular (HW = 15.3 mm; HL = 19.9 mm; HH = 8.7 mm) and neck distinct. Rostral scale rectangular, much wider than tall and as wide as mental. Nostrils separated from rostral by prenasals. Two enlarged prenasals in contact with rostral and first supralabials, both separated by a single scale. 9/9 smooth and distinctly enlarged supralabials, followed by two pairs of smooth smaller scales, followed by two keeled scales above the mouth commissure. Canthal ridges well developed with a distinct median depression. Scales covering canthal ridges, loreal, temporal and periphery of the parietal region distinctly enlarged and slightly keeled. Eyes desiccated (ED = 5.6 mm). Dorsal head pholidosis posterior to the eyes juxtaposed, similar in size to interorbital scales (distE = 3.1 mm). Ear opening is a vertical slit (EO = 2.8 mm). 9/9 smooth and distinctly enlarged infralabials. First three infralabials slightly larger than others on both sides. Mental bell-shaped, bordered posteriorly by a pair of elongated, large and hexagonal postmentals. Each postmental in contact with six scales: mental, one postmental, first infralabial, one enlarged lateral gular, one smaller posterolateral gular and one larger central gular. Other gulars small and juxtaposed.

Scales covering the dorsal side of neck heterogeneous, with enlarged, markedly-keeled scales regularly separated from each other transversally and longitudinally by a row of 2–3 small, smooth and juxtaposed scales. Scales covering the lateral side of neck small, smooth and juxtaposed. Dorsally, 19 rows of enlarged markedly-keeled scales counted transversally at mid-body, regularly separated from each other transversally and longitudinally by a row of 1–2 smaller, keeled and juxtaposed scales. Vertebral line with a single distinct row of smaller keeled scales. Thirty-three dorsal-enlarged keeled scales from nape to tail. Ventral scales of neck, chest and abdomen flat, roundish and slightly imbricated.

Original tail (TL = 49 mm), slightly flattened dorsoventrally. Tail scales mostly keeled and very spiny. Dorsal pygal scales similar to dorsal body scales, only slightly more prominent. Regular transverse rows (whorls) with eight very spiny pygal scales per whorl. The first three whorls, with lateral pygal scales smaller and keeled. After the third whorl, lateral scales smooth. Ventral pygal section of tail with a pair of postcloacal sacs. Ventral scales of the tail between spiny pygal scales small and flat.

Dorsal scales of forelimbs and hind limbs mostly keeled. Ventral scales of forelimbs slightly smaller than surrounding ventral scales of the body. Ventral scales of hind limbs similar in size to surrounding ventral scales of the body. Hands (HAL = 7.1 mm) and feet (FoL = 9.3 mm) with proximal subdigital scales in rows of mostly two. Digits distinctly expanded at tips. One pair of squarish terminal adhesive pads. Claws curving downwards between terminal pads of digits.

After nine years in ethanol (Fig. 2), the overall colouration of the preserved holotype is similar to the colouration in life (Fig. 8). Background colours of the holotype are ochre and light brown. Melanophores are present in both the ochre and light brown body areas and their different concentrations across the body define contrasting darker brown markings. Head dorsally overall ochre with irregular light brown markings and with densely packed melanophores that define the curly-bracket-shaped transverse stripe in the nuchal region. This continues to reach the eyes on both sides. Other densely-packed areas of melanophores define two light brown blotches behind the eyes and a central vacuity in the parietal region. Altogether, this pattern of densely packed melanophores define the “butterfly” pattern on the head. Pair of light brown lateral bands running from second supralabial to the anterior corner of the eyes and from the posterior corner of the eyes to the nuchal region, where they merge with the background light brown body colouration. Body dorsally light brown with six irregular ochre patches. These are delimited by a line of densely-packed melanophores, that borders each patch externally and sometimes internally, creating irregular darker brown markings. These patches are the following (from head to cloaca): one narrow on the neck and discontinuous at mid-body (3.3 mm width along vertebral axis at mid-body), one at the forelimb insertion (4.8 mm), one posterior to the forelimb insertion along the vertebral axis (3.1 mm), one distinctly broader bow-tie-shaped at mid-body (9.1 mm), one discontinuous anterior to the hind limbs (with the aspect of three longitudinal short bands not in contact, 4.3 mm) and a very distinct patch between the hind limbs (6.3 mm). Dorsal colouration fading to ventral colouration along body flanks (this includes the irregular ochre patches). Tail colour similar to dorsum, with twelve regularly-distanced ochre whorls. These are separated by light brown whorls defined by scales bordered by one or two stripes of densely-packed melanophores. Dorsal surfaces of forelimbs and hind limbs light brown with irregular dark markings determined by densely-packed melanophores that confer a marbled appearance. Ventral colouration (throat, chest, abdomen, ventral parts of forelimbs and hind limbs) ochre. Ventral scales generally with less than ten melanophores each, number decreasing towards mid-body. Infralabials (except the first on left side and except the first three on right side) and peripheral gulars on both sides of throat pigmented for at least one third of their surface, giving an overall brownish colour. Peripheral gular scales on both sides of throat light brown and with melanophores over at least one third of their surface. Ventral surface of the tail ochre with irregular dark markings determined by densely-packed melanophores that confer a marbled appearance. Scales of the ventral surfaces of hands and feet with densely-packed melanophores, giving a dark brown colouration.

##### Etymology.

The specific epithet is a noun in apposition to the genus name, derived from the Malagasy words “manonga” (ma-noon-ga) meaning “to climb”, and “vato” (va-too) meaning “rock”, because the species dwells on large granitic boulders. Additionally, the name evokes rock climbing, as the area, especially around Tsaranoro, has many well-known sites for this sport.

##### Natural history, habitat and distribution.

Based on genetically verified records, *Paroeduramanongavato* sp. nov. is known from two localities, Anja Reserve and Tsaranoro Valley Forest (Forêt Sacrée), which are separated by ca. 25 km and are located on the south-central plateau of Madagascar, south of the city of Ambalavao. The holotype was found at dusk active on a large boulder at the entrance of Anja Reserve. In Anja, other individuals of this species were found active both during day and night on granitic boulders within patches of semi-arid deciduous forest and in large cavities below these boulders, in a quite humid environment. The individuals ACZC10441 (ACP5940) and UADBA R-uncatalogued (ACZCV 0528; ACZC10442; ACP4725) were found at night moving on the walls of a cave-like recess created by large granitic boulders at ca. 1.5 m above the ground. ACZC10441 jumped on the leaf litter once spotted. The individuals UADBA R-uncatalogued (ACZCV 0782) and UADBA R-uncatalogued (ACZCV 0777) were found at night on boulders in the most internal part of Tsaranoro Forest. *Paroeduramanongavato* sp. nov. seems associated with granitic boulders within semi-arid deciduous forest and it occurs in close syntopy with the morphologically similar *P.rennerae*.

##### Conservation and proposed IUCN Red List status.

The Extent of Occurrence (EOO) and Area of Occupancy (AOO) computed for this species are 3.032 km^2^ and 12 km^2^, respectively. We propose a classification of *Paroeduramanongavato* sp. nov. as Critically Endangered (CR), under the criteria B1ab(iii) of the IUCN Red List guidelines (IUCN Standards and Petitions Committee 2022). This proposed evaluation is based on the narrow EOO, the severely fragmented habitat where the species is encountered and the observed decline in the extent and quality of the habitat.

## ﻿Discussion

Miralles et al. (2021) provided a taxonomic revision of the *Paroedurabastardi* clade, in which they identified a candidate species (*P.bastardi* D) that could not be formally described due to the limited amount of genetic data and the lack of available specimens. This candidate species is described in this study as *P.manongavato* sp. nov., increasing the total number of named species in the genus to 25. Our multilocus phylogenetic hypothesis placed the new species as sister to the *P.guibeae* lineage from Tranoroa (Fig. 6). *Paroeduraguibeae* is known to contain at least four mitochondrial lineages, two of which were considered as distinct candidate species by Cocca et al. (2018), in accordance with our results of high intraspecific uncorrected *p*-distance range (0.0–14.8%). A taxonomic assessment of the *Paroeduraguibeae* clade is, therefore, needed to clarify their species status.

*Paroeduramanongavato* sp. nov. is currently known from only two sites, where it is found living in close syntopy with the morphologically similar *P.rennerae*. Differently from *P.manongavato* sp. nov., *P.rennerae* has a wider distribution and apparently a broader substrate use, with records from granitic boulders in forested areas, caves, human settlements, tree trunks (Kirindy) and inside canyons (Isalo: Zahavola) (Miralles et al. 2021). In contrast, we only found records of *P.manongavato* sp. nov. from boulders inside patches of semi-arid deciduous forest, indicating that this species likely depends on forest. The lack of gene flow in the analysed nuclear genes between these two species from the same localities indicates the presence of reproductive isolation between them and suggests that other intrinsic or extrinsic factors could be involved in their diversification. For example, the difference in size between the two species might play a role in their substrate use, with *P.manongavato* sp. nov. preferring a rocky habitat and possibly occupying different positions on the surfaces or cavities of granitic boulders compared with *P.rennerae*. Additional field data are needed to characterise their differential niche use where they are found in syntopy.

The severe deforestation over the last seven decades in Madagascar (Harper et al. 2007; Vieilledent et al. 2018) has led to a dramatically fragmented landscape of the south-central plateau of Madagascar (between Fianarantsoa and Andringitra National Park; Cameron and Gould (2013)). Repeated forest clearings through slash-and-burn have accelerated soil erosion and impacted the soil water retention, leading to an impoverishment of land fertility in many areas of Madagascar, including the south-central plateau (Palm et al. 1996; Styger et al. 2007; Scales 2011; Gould and Andrianomena 2015). It is likely that local taboos (fady) on ancestral burial places (i.e. ancient Betsileo tombs) provided shelter for some forest sites in the area (Bodin et al. 2006; Gould and Andrianomena 2015), including Anja and Tsaranoro. *Paroeduramanongavato* sp. nov. occurs in a severely fragmented landscape, made of isolated suitable forest patches surrounded by anthropogenically-modified and unsuitable habitats, including agricultural field, pastures, grasslands, human settlements and roads (Cameron and Gould 2013; Gould and Gabriel 2015; Gould and Cowen 2020). In this landscape, Anja Reserve and Tsaranoro are the two largest semi-arid deciduous forest fragments of the south-central plateau, of 36 ha and 46 ha, respectively (Gould and Andrianomena 2015). Smaller forest remnants are present nearby, i.e. Sakaviro (14 ha, 8 km N of Anja Reserve) and Ambatomainty (ca. 2 ha, 9 km N of Tsaranoro), but no records of *P.manongavato* sp. nov. are known from these fragments (Belluardo et al. 2021), suggesting that this species may require specific canopy cover and forest structure to maintain viable populations.

Anja Reserve and Tsaranoro are isolated patches outside the network of legally-protected areas of Madagascar (Goodman et al. 2018). At the same time, these sites are considered to have high cultural and touristic interest, due to the abundance of hiking trails, climbing sites and populations of the ring-tailed lemur (*Lemurcatta* Linnaeus, 1758), the only primate inhabiting these forest patches (Cameron and Gould 2013; Gould and Andrianomena 2015; Gould and Gabriel 2015). Anja’s potential as an ecotourism site led to its designation as a community-managed reserve in 1999. Local community associations Anja Miray (in 2000) and Tantely (in 2002) were established to respectively manage Anja Reserve and Tsaranoro Valley Forest, in response to selective logging of native trees, the increasing forest clearance by slash-and-burn actions and consequent impoverishment of land fertility and loss of agricultural profit (Gould and Andrianomena 2015; Gabriel et al. 2018). Both associations have been active in protecting these forests, using the incomes of ecotourism activities and donations (United Nations Development Programme 2013). In Tsaranoro, funds were used to establish tree nurseries aiming to expand the forest surface and to build breaks against uncontrolled grassfire set in pastures (Gould and Andrianomena 2015). Despite these efforts, it will take several years to mitigate the effects of the past and ongoing deforestation for agriculture, firewood and pastures in the surroundings of Anja Reserve (Crottini et al. 2015) and Tsaranoro (Gould and Andrianomena 2015; Gould and Gabriel 2015). The conservation value of these sites is remarkable. Recent cataloguing efforts of amphibian and reptile species have highlighted the high herpetological diversity of several forest fragments surrounding the Andringitra Massif (Belluardo et al. 2021), with several recently-described species only known from a single or a few sites in the area (Crottini et al. 2011, 2012b, 2015). The management of these sites is key to ensuring the survival of such forest fragments, developing local ecotourism, creating job opportunities for villagers and promoting a more sustainable land use (Gould and Andrianomena 2015). This is essential for the survival of the several microendemic species that are found in these forest fragments (Jenkins et al. 2014).

Many Malagasy gecko species have a narrow distribution range, with several species recorded only from their type locality (Meiri et al. 2018). This is reflected in their conservation status, with one-third of Malagasy geckos belonging to one of the IUCN threatened categories (Jenkins et al. 2014). With an extent of occurrence of ca. 3 km^2^ and records in only two isolated forest fragments, *P.manongavato* sp. nov. ranked as Critically Endangered (CR) in our assessment (IUCN Standards and Petitions Committee 2022). As habitat loss and fragmentation continue to increase in the southern-central plateau, the conservation value of these forest patches becomes increasingly significant. Considering that reptiles have high ecotouristic potential in Madagascar (Wollenberg et al. 2011), the presence of these microendemic reptile species as site-specific attractions can hold substantial value. This is true not only in terms of economic income and development for the local communities, that can invest the monetary revenue into training guides and supporting habitat preservation initiatives (e.g. the tree nurseries in Tsaranoro, Gould and Andrianomena (2015)), but also in terms of conservation education. By raising awareness on the protection importance of Malagasy biodiversity, tourism can help promote conservation efforts while providing a sustainable source of income for local communities.

Species inventories and taxonomic research are fundamental to improve our knowledge on global biodiversity and remain of paramount importance in advancing species’ conservation status assessments. This is especially true for narrow-ranged species, which have a higher extinction risk. Our study contributed to this global effort with the naming of a new microendemic species of the genus *Paroedura*.

## Supplementary Material

XML Treatment for Paroedura manongavato

## ﻿Additional information

### Author ORCIDs

Costanza Piccoli https://orcid.org/0000-0002-1234-5706

Francesco Belluardo https://orcid.org/0000-0002-3967-2686

Javier Lobón-Rovira https://orcid.org/0000-0003-4380-9427

Ivo Oliveira Alves https://orcid.org/0009-0000-1187-4211

Malalatiana Rasoazanany https://orcid.org/0009-0008-4242-8191

Franco Andreone https://orcid.org/0000-0001-9809-5818

Gonçalo M. Rosa https://orcid.org/0000-0002-8658-8436

Angelica Crottini https://orcid.org/0000-0002-8505-3050

### Data availability

All of the data that support the findings of this study are available in the main text or Supplementary Information.

## Supplementary materials

Supplementary material 1 List of specimens and samples used in the molecular analyses Data type xlsx Explanation note List of specimens and samples used in the molecular analyses, including information on their locality, sex/stage, field, catalogue, tissue and extraction numbers and GenBank accession numbers. Underlined coordinates were extracted from the toponym using Google Earth. Accession numbers in bold are of newly-generated sequences. NA: information not available. **1A** molecular data used in **Dataset 1** (16S uncorrected *p*-distance), **Dataset 2** (COI ML tree), **Datasets 3 and 4** (KIAA1239 and CMOS haplotype network reconstruction, including the respective haplotype numbers). Sequences marked with asterisk were not used in the haplotype network analyses. **1B** molecular data used in **Dataset 5** (multilocus phylogenetic analysis). Sequences for nuclear genes ACM4, MXRA5 and PRLR were extracted from the full genome of *Paroedurapicta* (Hara et al. 2018): MXRA5: BDOT02000005.1:77606075-77606865; ACM4: BDOT02000002.1:110684587-110684921; PRLR: BDOT02000007.1:89098037-89098524. https://binary.pensoft.net/file/916542This dataset is made available under the Open Database License (http://opendatacommons.org/licenses/odbl/1.0/). The Open Database License (ODbL) is a license agreement intended to allow users to freely share, modify, and use this Dataset while maintaining this same freedom for others, provided that the original source and author(s) are credited.Costanza Piccoli, Francesco Belluardo, Javier Lobón-Rovira, Ivo Oliveira Alves, Malalatiana Rasoazanany, Franco Andreone, Gonçalo M. Rosa, Angelica Crottini

Supplementary material 2 Scales counts and measures collected with ImageJ from photographic material Data type docx Explanation note Scales counts and measures collected with ImageJ from photographic material of genetically-determined specimens of *Paroeduramanongavato* sp. nov. (ACP5940; ACP4725; ACP4991). https://binary.pensoft.net/file/917299This dataset is made available under the Open Database License (http://opendatacommons.org/licenses/odbl/1.0/). The Open Database License (ODbL) is a license agreement intended to allow users to freely share, modify, and use this Dataset while maintaining this same freedom for others, provided that the original source and author(s) are credited.Costanza Piccoli, Francesco Belluardo, Javier Lobón-Rovira, Ivo Oliveira Alves, Malalatiana Rasoazanany, Franco Andreone, Gonçalo M. Rosa, Angelica Crottini

Supplementary material 3 Best-fit substitution models and best partitioning scheme calculated using Partition Finder 2.1.1 Data type docx https://binary.pensoft.net/file/917300This dataset is made available under the Open Database License (http://opendatacommons.org/licenses/odbl/1.0/). The Open Database License (ODbL) is a license agreement intended to allow users to freely share, modify, and use this Dataset while maintaining this same freedom for others, provided that the original source and author(s) are credited.Costanza Piccoli, Francesco Belluardo, Javier Lobón-Rovira, Ivo Oliveira Alves, Malalatiana Rasoazanany, Franco Andreone, Gonçalo M. Rosa, Angelica Crottini

Supplementary material 4 List of haplotypes Data type docx Explanation note List of haplotypes inferred by DnaSP 6.12.03 (PHASE algorithm) and used in the haplotype network analyses of nuclear genes **A** KIAA1239, **B**CMOS. https://binary.pensoft.net/file/916545This dataset is made available under the Open Database License (http://opendatacommons.org/licenses/odbl/1.0/). The Open Database License (ODbL) is a license agreement intended to allow users to freely share, modify, and use this Dataset while maintaining this same freedom for others, provided that the original source and author(s) are credited.Costanza Piccoli, Francesco Belluardo, Javier Lobón-Rovira, Ivo Oliveira Alves, Malalatiana Rasoazanany, Franco Andreone, Gonçalo M. Rosa, Angelica Crottini

Supplementary material 5 Morphological dataset Data type docx Explanation note Morphological dataset used in the analysis of the phenotypic variation in the *Paroedurabastardi* clade. https://binary.pensoft.net/file/917301This dataset is made available under the Open Database License (http://opendatacommons.org/licenses/odbl/1.0/). The Open Database License (ODbL) is a license agreement intended to allow users to freely share, modify, and use this Dataset while maintaining this same freedom for others, provided that the original source and author(s) are credited.Costanza Piccoli, Francesco Belluardo, Javier Lobón-Rovira, Ivo Oliveira Alves, Malalatiana Rasoazanany, Franco Andreone, Gonçalo M. Rosa, Angelica Crottini
