# Supplementary material for: ﻿Another step through the crux: a new microendemic rock-dwelling Paroedura (Squamata, Gekkonidae) from south-central Madagascar
Source: Zookeys. 2023 Oct 4;1181:125–54. doi: 10.3897/zookeys.1181.108134 (PMC10568478; doi:10.3897/zookeys.1181.108134)
Supplement: Supplementary material 2 — Scales counts and measures collected with ImageJ from photographic material [file zookeys-1181-125_article-108134__-s002.docx]

Appendix 2. Scales counts and measures collected with ImageJ from photographic material of genetically-determined specimens of *Paroedura* *manongavato* **sp. nov.** (ACP5940; ACP4725; ACP4991).

Appendix 2.1. Dorsal scales longitudinal counts (character **DorL**, three repetitions) from photographic material of genetically-determined specimens of *Paroedura manongavato* **sp. nov.** (first row: ACP5940; second row: ACP4725; third row: ACP4991). Photographs by JLR.


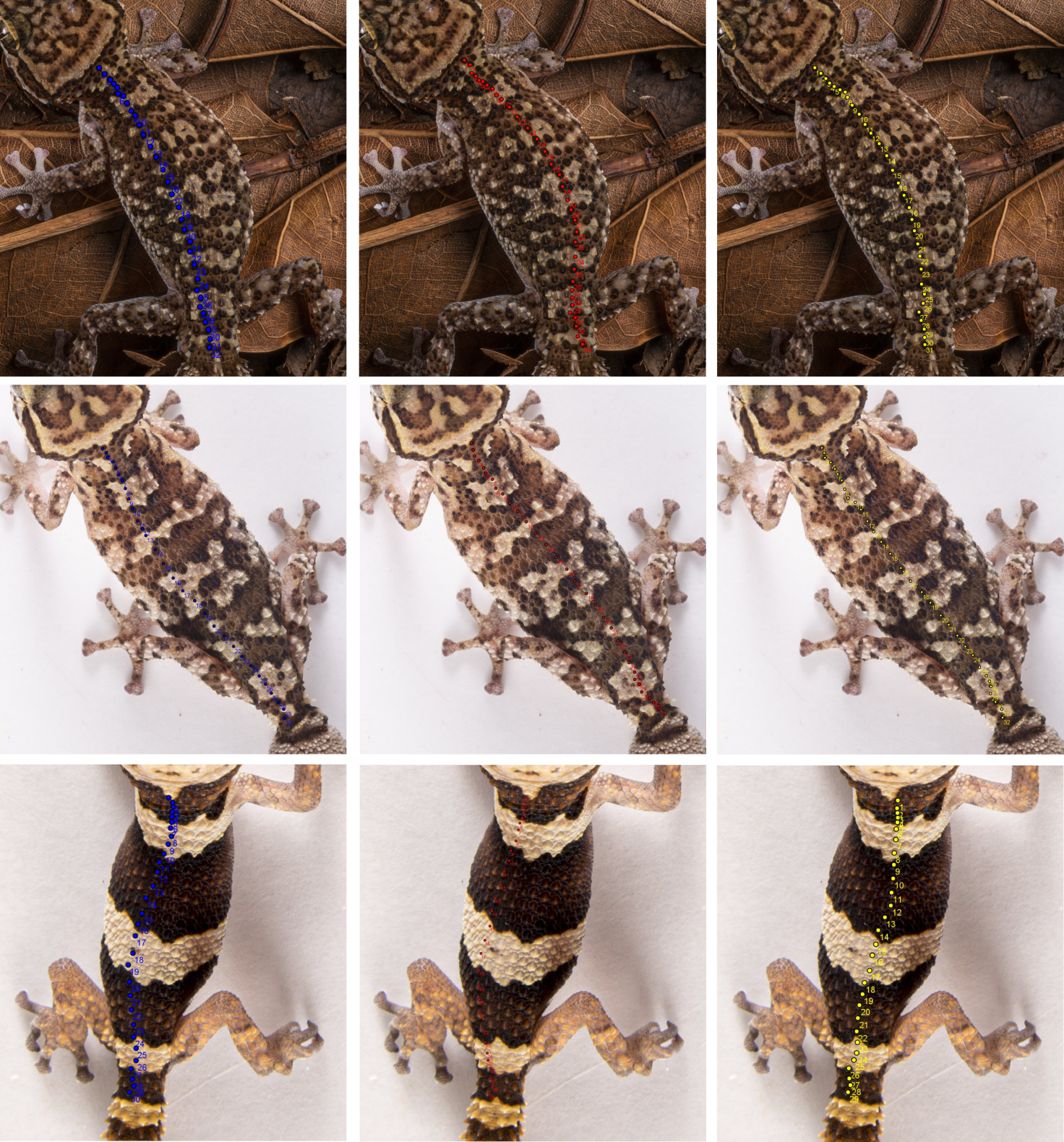


Appendix 2.2. Interorbital scales counts (character **IO**) from photographic material of genetically-determined specimens of *Paroedura manongavato* **sp. nov.** (first row: ACP5940 and ACP4725; second row: ACP2761 (holotype) and ACP4991). Photographs of ACP5940, ACP4725 and ACP4991 by JLR; photogtaph of ACP2761 by CP.


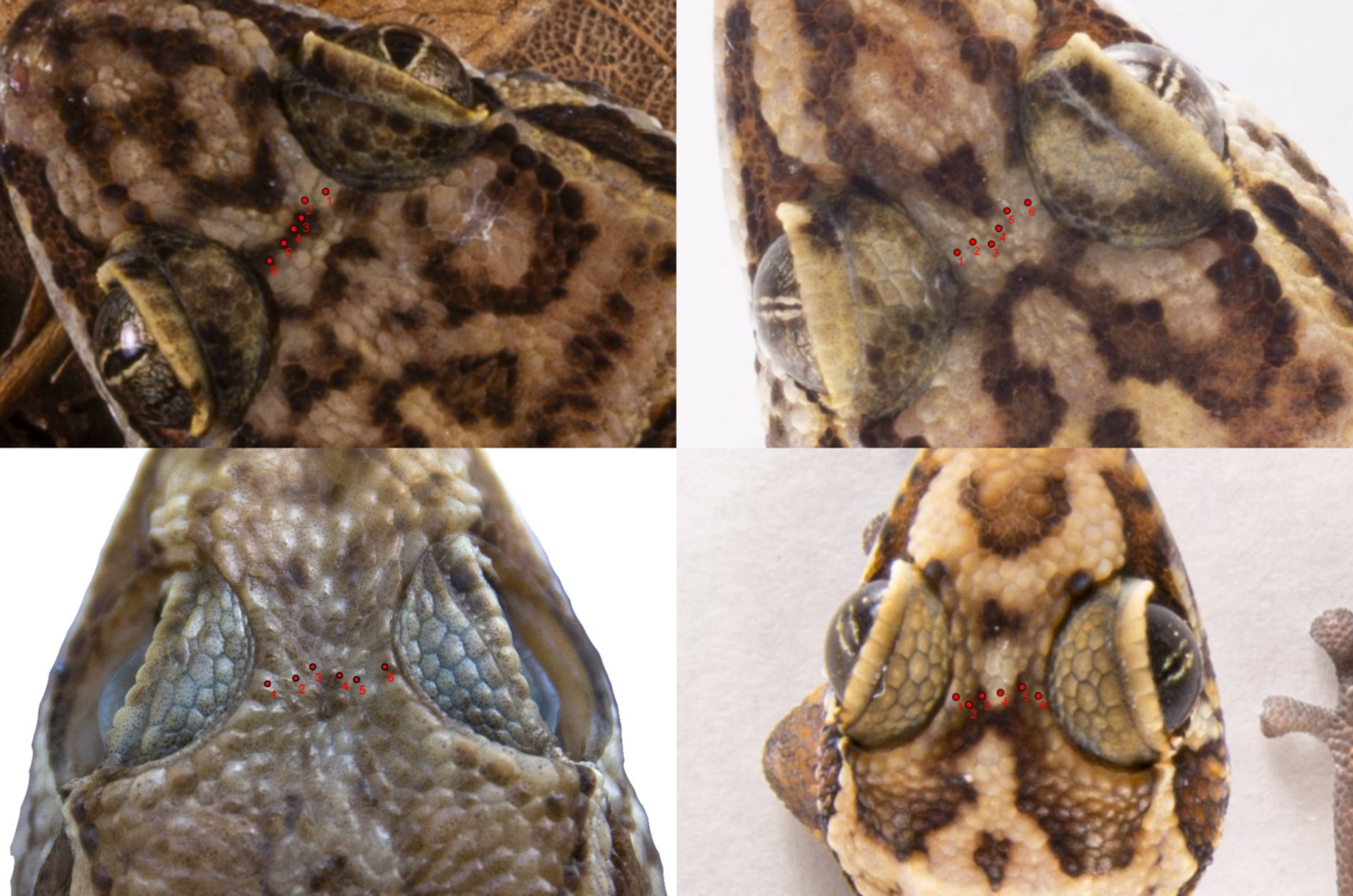


Appendix 2.3. Snout tip scales counts (character **SeP**) from photographic material of genetically-determined specimens of *Paroedura manongavato* **sp. nov.** (first picture: ACP5940; second picture: ACP4725; third picture: ACP4991). Photographs by JLR.


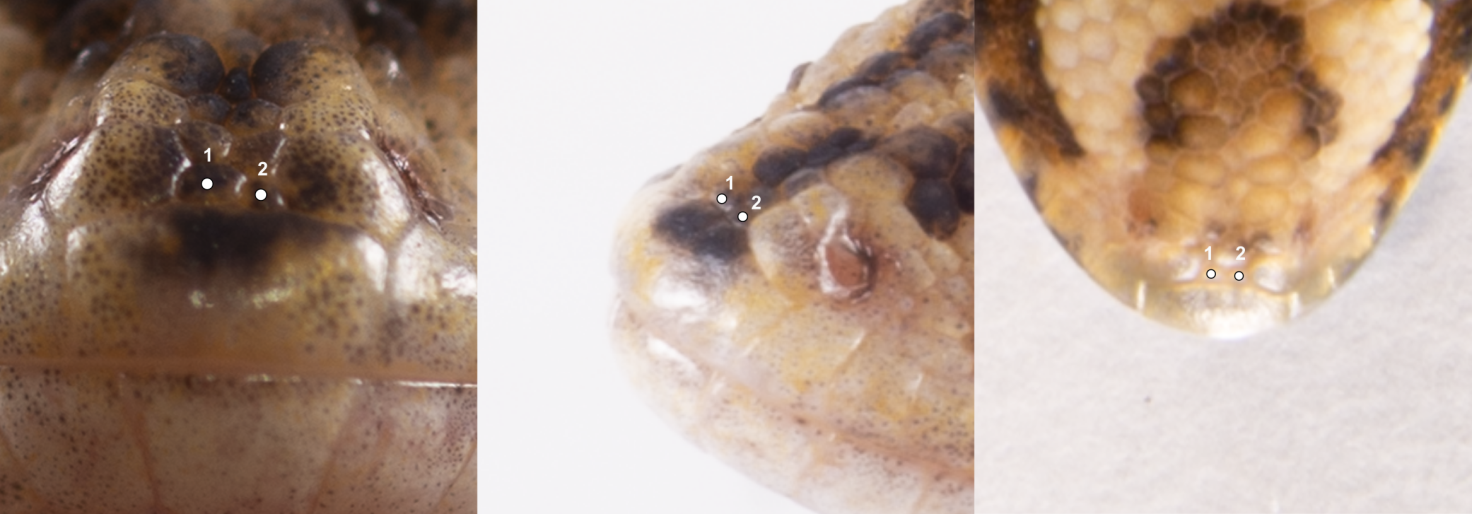


Appendix 2.4. Measures of specimen ACP4725. Characters: **SVL**, snout–vent length; **TL**, tail length; **HL**, head length from the anterior margin of the ear opening to the tip of the snout; **HW**, maximum head width; **distE**, minimum distance between the bony edges of the orbits in dorsal view; **ED**, maximum eye diameter; **FoL**, foot length (from ankle to tip of the 3^th^ toe, left side); **HAL**, hand length, distance between the wrist and the tip of the longest finger (until the insertion of the claw, which is not included, left side). Photographs by JLR.


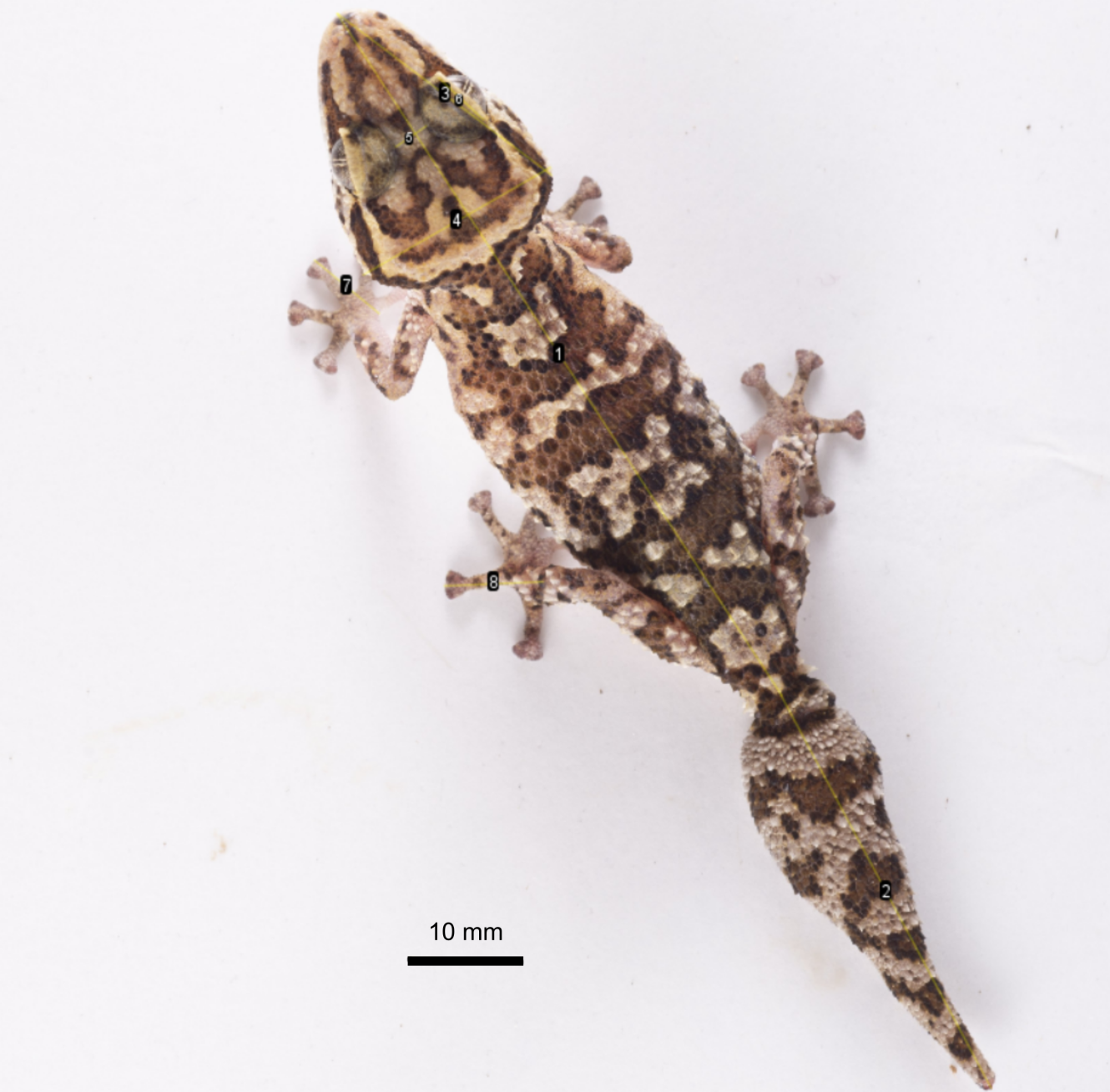


|  | Measure | Length (mm) |
| --- | --- | --- |
| 1 | SVL | 73.024 |
| 2 | TL | 40.01 |
| 3 | HW | 24.042 |
| 4 | HL | 19.281 |
| 5 | distE | 3.275 |
| 6 | ED | 5.421 |
| 7 | HAL | 7.45 |
| 8 | FoL | 8.857 |
