# Supplementary material for: ﻿Another step through the crux: a new microendemic rock-dwelling Paroedura (Squamata, Gekkonidae) from south-central Madagascar
Source: Zookeys. 2023 Oct 4;1181:125–54. doi: 10.3897/zookeys.1181.108134 (PMC10568478; doi:10.3897/zookeys.1181.108134)
Supplement: Supplementary material 3 — Best-fit substitution models and best partitioning scheme calculated using Partition Finder 2.1.1 [file zookeys-1181-125_article-108134__-s003.docx]

Appendix 3. Best-fit substitution models and best partitioning scheme calculated using Partition Finder 2.1.1.

| Settings used |
| --- |
| branchlengths: linked |
| models: JC, K80, SYM, F81, HKY, GTR, JC+G, K80+G, SYM+G, F81+G, HKY+G, GTR+G, JC+I, K80+I, SYM+I, F81+I, HKY+I, GTR+I, JC+I+G, K80+I+G, SYM+I+G, F81+I+G, HKY+I+G, GTR+I+G |
| model_selection: aicc |
| search: greedy |

| Best partitioning scheme |
| --- |
| Scheme Name: step_20 |
| Scheme lnL: -25706.09144592285 |
| Scheme AICc: 51714.5151268 |
| Number of params: 149 |
| Number of sites: 10468 |
| Number of subsets: 19 |

| Subset | Best Model | # sites | Partition names |
| --- | --- | --- | --- |
| 1 | GTR+I+G | 1286 | tRNA, 16S, 12S |
| 2 | HKY+G | 417 | ND4_pos2, ND2_pos2 |
| 3 | GTR+G | 417 | ND2_pos3, ND4_pos3 |
| 4 | GTR+I | 192 | ND2_pos1 |
| 5 | GTR+G | 224 | ND4_pos1 |
| 6 | SYM+G | 206 | COXI_pos1 |
| 7 | F81+I+G | 206 | COXI_pos2 |
| 8 | HKY+G | 206 | COXI_pos3 |
| 9 | F81+I | 397 | ACM4_pos1, TTN_pos1 |
| 10 | F81+I | 114 | ACM4_pos2 |
| 11 | K80+G | 114 | ACM4_pos3 |
| 12 | HKY | 1453 | RAG1_pos1, CMOS_pos1, SACS_pos1, MXRA5_pos1 |
| 13 | HKY+I | 854 | TTN_pos2, PDC_pos2, CMOS_pos2, KIAA1239_pos2 |
| 14 | GTR+G | 919 | PRLR_pos2, RAG1_pos3, MXRA5_pos3, CMOS_pos3 |
| 15 | HKY+G | 427 | PDC_pos3, KIAA1239_pos3 |
| 16 | F81+I | 425 | PDC_pos1, KIAA1239_pos1 |
| 17 | GTR+G | 1308 | RAG1_pos2, MXRA5_pos2, SACS_pos2 |
| 18 | HKY+I | 325 | PRLR_pos1, PRLR_pos3 |
| 19 | GTR+G | 978 | SACS_pos3, TTN_pos3 |
