# Supplementary material for: ﻿Another step through the crux: a new microendemic rock-dwelling Paroedura (Squamata, Gekkonidae) from south-central Madagascar
Source: Zookeys. 2023 Oct 4;1181:125–54. doi: 10.3897/zookeys.1181.108134 (PMC10568478; doi:10.3897/zookeys.1181.108134)
Supplement: Supplementary material 4 — List of haplotypes [file zookeys-1181-125_article-108134__-s004.docx]

Appendix 4. List of haplotypes inferred by DnaSP 6.12.03 (PHASE algorithm) and used in the haplotype network analyses of nuclear genes **A** KIAA1239, **B** CMOS.

| (**A**) KIAA1239 | | | | |
| --- | --- | --- | --- | --- |
| Haplotype  number | Number of sequences | Species sequence | Voucher/Tissue | Locality |
| Hap_1 | 27 | 1.Prennerae-1  2.Prennerae-1  2.Prennerae-2  3.Prennerae-1  3.Prennerae-2  4.Prennerae-1  5.Prennerae-1  6.Prennerae-1  6.Prennerae-2  7.Prennerae-1  7.Prennerae-2  8.Prennerae-1  8.Prennerae-2  9.Prennerae-1  9.Prennerae-2  17.Prennerae-1  17.Prennerae-2  22.Prennerae-1  22.Prennerae-2  23.Prennerae-1  23.Prennerae-2  24.Prennerae-1  24.Prennerae-2  26.Prennerae-1  26.Prennerae-2  28.Prennerae-1  28.Prennerae-2 | MirZC040  MirZC047  MirZC047  MirZC091  MirZC091  MirZC101  MirZC102  MirZC103  MirZC103  MirZC104  MirZC104  ZSM 0849/2010 (ZCMV12740)  ZSM 0849/2010 (ZCMV12740)  ZCMV12754  ZCMV12754  ACZCV 0745  ACZCV 0745  ACZC10911  ACZC10911  ACZCV 0764  ACZCV 0764  ACZCV 0804  ACZCV 0804  ACZCV 0806  ACZCV 0806  ACZC10998  ACZC10998 | Kirindy  Kirindy  Kirindy  Kirindy  Kirindy  Kirindy  Kirindy  Kirindy  Kirindy  Kirindy  Kirindy  Kirindy  Kirindy  Kirindy  Kirindy  Sakaviro  Sakaviro  Sakaviro  Sakaviro  Tsaranoro  Tsaranoro  Ambatomainty  Ambatomainty  Ambatomainty  Ambatomainty  Ambatomainty  Ambatomainty |
| Hap_2 | 1 | 1.Prennerae-2 | MirZC040 | Kirindy |
| Hap_3 | 1 | 4.Prennerae-2 | MirZC101 | Kirindy |
| Hap_4 | 1 | 5.Prennerae-2 | MirZC102 | Kirindy |
| Hap_5 | 6 | 10.Prennerae-1  10.Prennerae-2  11.Prennerae-1  11.Prennerae-2  14.Prennerae-1  14.Prennerae-2 | ZCMV12789  ZCMV12789  ZCMV12791  ZCMV12791  ACZCV 0526  ACZCV 0526 | Anja Reserve  Anja Reserve  Anja Reserve  Anja Reserve  Anja Reserve  Anja Reserve |
| Hap_6 | 1 | 12.Prennerae-1 | ACZCV 0524 | Anja Reserve |
| Hap_7 | 1 | 12.Prennerae-2 | ACZCV 0524 | Anja Reserve |
| Hap_8 | 1 | 13.Prennerae-1 | ACZCV 0525 | Anja Reserve |
| Hap_9 | 1 | 13.Prennerae-2 | ACZCV 0525 | Anja Reserve |
| Hap_10 | 3 | 15.Prennerae-1  15.Prennerae-2  16.Prennerae-1 | ACZC6534 (FAZC14743)  ACZC6534 (FAZC14743)  ACZC6438 (FAZC14631) | Isalo  Isalo  Isalo |
| Hap_11 | 1 | 16.Prennerae-2 | ACZC6438 (FAZC14631) | Isalo |
| Hap_12 | 1 | 18.Prennerae-1 | ACZCV 0746 | Sakaviro |
| Hap_13 | 2 | 18.Prennerae-2  21.Prennerae-2 | ACZCV 0746  ACZC10906 | Sakaviro  Sakaviro |
| Hap_14 | 1 | 19.Prennerae-1 | ACZC10892 | Sakaviro |
| Hap_15 | 1 | 19.Prennerae-2 | ACZC10892 | Sakaviro |
| Hap_16 | 1 | 20.Prennerae-1 | ACZC10899 | Sakaviro |
| Hap_17 | 1 | 20.Prennerae-2 | ACZC10899 | Sakaviro |
| Hap_18 | 5 | 21.Prennerae-1  25.Prennerae-1  25.Prennerae-2  27.Prennerae-1  27.Prennerae-2 | ACZC10906  ACZCV 0805  ACZCV 0805  ACZC10994  ACZC10994 | Sakaviro  Ambatomainty  Ambatomainty  Ambatomainty  Ambatomainty |
| Hap_19 | 2 | 29.PlinD-1  29.PlinD-2 | ZCMV12790  ZCMV12790 | Anja Reserve  Anja Reserve |
| Hap_20 | 3 | 30.PlinD-1  32.PlinD-1  32.PlinD-2 | ACZCV 0528  ACZC1930  ACZC1930 | Anja Reserve  Anja Reserve  Anja Reserve |
| Hap_21 | 1 | 30.PlinD-2 | ACZCV 0528 | Anja Reserve |
| Hap_22 | 1 | 31.PlinD-1 | ACZCV 0300 | Anja Reserve |
| Hap_23 | 1 | 31.PlinD-2 | ACZCV 0300 | Anja Reserve |
| Hap_24 | 1 | 33.PlinD-1 | ACZC10441 | Anja Reserve |
| Hap_25 | 1 | 33.PlinD-2 | ACZC10441 | Anja Reserve |
| Hap_26 | 1 | 34.PlinD-1 | ACZCV 0777 | Tsaranoro |
| Hap_27 | 1 | 34.PlinD-2 | ACZCV 0777 | Tsaranoro |
| Hap_28 | 1 | 35.PlinD-1 | ACZCV 0782 | Tsaranoro |
| Hap_29 | 1 | 35.PlinD-2 | ACZCV 0782 | Tsaranoro |
| Hap_30 | 6 | 36.Pbastardi-1  36.Pbastardi-2  37.Pbastardi-1  37.Pbastardi-2  38.Pbastardi-1  38.Pbastardi-2 | ZSM 0178/2004 (FGZC0327)  ZSM 0178/2004 (FGZC0327)  ZSM 0180/2004 (FGZC0332)  ZSM 0180/2004 (FGZC0332)  FGMV2002.B47  FGMV2002.B47 | Tranoroa  Tranoroa  Tranoroa  Tranoroa  Tolagnaro  Tolagnaro |
| Hap_31 | 1 | 39.Pbastardi-1 | ZSM 0042/2004 (FGZC0070) | Tolagnaro |
| Hap_32 | 1 | 39.Pbastardi-2 | ZSM 0042/2004 (FGZC0070) | Tolagnaro |
| Hap_33 | 2 | 40.Pbastardi-1  40.Pbastardi-2 | FGZC0313  FGZC0313 | Tolagnaro  Tolagnaro |
| Hap_34 | 4 | 41.Pguibeae-1  41.Pguibeae-2  42.Pguibeae-1  42.Pguibeae-2 | ACZC1828  ACZC1828  ACZC6464 (FAZC14657)  ACZC6464 (FAZC14657) | Isalo  Isalo  Isalo  Isalo |
| Hap_35 | 2 | 43.Pguibeae-1  43.Pguibeae-2 | MRSN R2529  MRSN R2529 | Isalo  Isalo |
| Hap_36 | 2 | 44.Pguibeae-1  44.Pguibeae-2 | MRSN R2568  MRSN R2568 | Isalo  Isalo |
| Hap_37 | 5 | 45.Pguibeae-1  46.Pguibeae-1  48.Pguibeae-1  49.Pguibeae-1  49.Pguibeae-2 | FGMV2002.1523  FGMV2002.1524  FGMV2002.1577  ZSM 1101/2003 (FGMV2002.1600)  ZSM 1101/2003 (FGMV2002.1600) | Toliara  Toliara  Toliara  Toliara  Toliara |
| Hap_38 | 1 | 45.Pguibeae-2 | FGMV2002.1523 | Toliara |
| Hap_39 | 1 | 46.Pguibeae-2 | FGMV2002.1524 | Toliara |
| Hap_40 | 1 | 47.Pguibeae-1 | FGMV2002.1576 | Toliara |
| Hap_41 | 1 | 47.Pguibeae-2 | FGMV2002.1576 | Toliara |
| Hap_42 | 1 | 48.Pguibeae-2 | FGMV2002.1577 | Toliara |
| Hap_43 | 4 | 50.Pguibeae-1  50.Pguibeae-2  51.Pguibeae-1  51.Pguibeae-2 | ZSM 0187/2004 (FGZC0352)  ZSM 0187/2004 (FGZC0352)  ZSM 0188/2004 (FGZC0353)  ZSM 0188/2004 (FGZC0353) | Tranoroa  Tranoroa  Tranoroa  Tranoroa |
| Hap_44 | 2 | 52.Pguibeae-1  52.Pguibeae-2 | ZSM 0189/2004 (FGZC0354)  ZSM 0189/2004 (FGZC0354) | Tranoroa  Tranoroa |
| Hap_45 | 9 | 53.Pibityensis-1  54.Pibityensis-1  54.Pibityensis-2  55.Pibityensis-1  55.Pibityensis-2  56.Pibityensis-1  56.Pibityensis-2  57.Pibityensis-1  57.Pibityensis-2 | FGMV2001.D37  FGMV2002.0990  FGMV2002.0990  FGMV2002.0991  FGMV2002.0991  FGMV2002.0992  FGMV2002.0992  FGMV2002.0993  FGMV2002.0993 | Itremo  Ibity  Ibity  Ibity  Ibity  Ibity  Ibity  Ibity  Ibity |
| Hap_46 | 1 | 53.Pibityensis-2 | FGMV2001.D37 | Itremo |
| Hap_47 | 11 | 58.Ptanjaka-1  58.Ptanjaka-2  59.Ptanjaka-1  59.Ptanjaka-2  64.Ptanjaka-1  64.Ptanjaka-2  65.Ptanjaka-1  66.Ptanjaka-2  67.Ptanjaka-2  70.Ptanjaka-2  72.Ptanjaka-1 | ZSM 36/2006 (FGZC0742)  ZSM 36/2006 (FGZC0742)  ZSM 40/2006 (FGZC0750)  ZSM 40/2006 (FGZC0750)  FGZC0967  FGZC0967  FGZC0968  ZSM 149/2006 (FGZC0969)  FGZC0753  ZSM 142/2006 (FGZC0950)  FGZC0952 | Bemaraha  Bemaraha  Bemaraha  Bemaraha  Bemaraha  Bemaraha  Bemaraha  Bemaraha  Bemaraha  Bemaraha  Bemaraha |
| Hap_48 | 2 | 60.Ptanjaka-1  60.Ptanjaka-2 | ZSM 53/2006 (FGZC0780)  ZSM 53/2006 (FGZC0780) | Bemaraha  Bemaraha |
| Hap_49 | 6 | 61.Ptanjaka-1  61.Ptanjaka-2  62.Ptanjaka-1  62.Ptanjaka-2  66.Ptanjaka-1  69.Ptanjaka-1 | FGZC0953  FGZC0953  FGZC0672  FGZC0672  ZSM 149/2006 (FGZC0969)  ZSM 43/2006 (FGZC0755) | Bemaraha  Bemaraha  Bemaraha  Bemaraha  Bemaraha  Bemaraha |
| Hap_50 | 1 | 63.Ptanjaka-1 | FGZC0717 | Bemaraha |
| Hap_51 | 1 | 63.Ptanjaka-2 | FGZC0717 | Bemaraha |
| Hap_52 | 4 | 65.Ptanjaka-2  68.Ptanjaka-1  69.Ptanjaka-2  72.Ptanjaka-2 | FGZC0968  FGZC0754  ZSM 43/2006 (FGZC0755)  FGZC0952 | Bemaraha  Bemaraha  Bemaraha  Bemaraha |
| Hap_53 | 3 | 67.Ptanjaka-1  70.Ptanjaka-1  71.Ptanjaka-1 | FGZC0753  ZSM 142/2006 (FGZC0950)  ZSM 143/2006 (FGZC0951) | Bemaraha  Bemaraha  Bemaraha |
| Hap_54 | 1 | 68.Ptanjaka-2 | FGZC0754 | Bemaraha |
| Hap_55 | 1 | 71.Ptanjaka-2 | ZSM 143/2006 (FGZC0951) | Bemaraha |
| Hap_56 | 1 | 73.Pneglecta-1 | ZSM 18/2006 (FGZC0704) | Bemaraha |
| Hap_57 | 3 | 73.Pneglecta-2  74.Pneglecta-1  75.Pneglecta-1 | ZSM 18/2006 (FGZC0704)  ZSM 163/2006 (FGZC0991)  ZSM 128/2006 (FGZC0992) | Bemaraha  Bemaraha  Bemaraha |
| Hap_58 | 2 | 74.Pneglecta-2  75.Pneglecta-2 | ZSM 163/2006 (FGZC0991)  ZSM 128/2006 (FGZC0992) | Bemaraha  Bemaraha |
| (**B**) CMOS | | | | |
| Haplotype  number | Number of sequences | Species sequence | Voucher/Tissue | Locality |
| Hap_1 | 35 | 1.Prennerae-1  1.Prennerae-2  3.Prennerae-1  3.Prennerae-2  4.Prennerae-1  4.Prennerae-2  5.Prennerae-1  5.Prennerae-2  7.Prennerae-1  7.Prennerae-2  8.Prennerae-1  8.Prennerae-2  12.Prennerae-1  12.Prennerae-2  13.Prennerae-1  14.Prennerae-1  17.Prennerae-1  17.Prennerae-2  18.Prennerae-1  18.Prennerae-2  20.Prennerae-1  25.Prennerae-1  25.Prennerae-2  26.Prennerae-1  26.Prennerae-2  27.Prennerae-1  27.Prennerae-2  28.Prennerae-1  28.Prennerae-2  29.Prennerae-1  29.Prennerae-2  30.Prennerae-1  30.Prennerae-2  31.Prennerae-1  31.Prennerae-2 | MirZC040  MirZC040  MirZC091  MirZC091  MirZC101  MirZC101  MirZC102  MirZC102  MirZC104  MirZC104  ZSM 0849/2010 (ZCMV12740)  ZSM 0849/2010 (ZCMV12740)  ACZCV 0524  ACZCV 0524  ACZCV 0525  ACZCV 0526  ACZC6534 (FAZC14743)  ACZC6534 (FAZC14743)  ACZC6438 (FAZC14631)  ACZC6438 (FAZC14631)  ACZCV 0746  ACZCV 0761  ACZCV 0761  ACZCV 0764  ACZCV 0764  ACZCV 0804  ACZCV 0804  ACZCV 0805  ACZCV 0805  ACZCV 0806  ACZCV 0806  ACZC10994  ACZC10994  ACZC10998  ACZC10998 | Kirindy  Kirindy  Kirindy  Kirindy  Kirindy  Kirindy  Kirindy  Kirindy  Kirindy  Kirindy  Kirindy  Kirindy  Anja Reserve  Anja Reserve  Anja Reserve  Anja Reserve  Isalo  Isalo  Isalo  Isalo  Sakaviro  Tsaranoro  Tsaranoro  Tsaranoro  Tsaranoro  Ambatomainty  Ambatomainty  Ambatomainty  Ambatomainty  Ambatomainty  Ambatomainty  Ambatomainty  Ambatomainty  Ambatomainty  Ambatomainty |
| Hap_2 | 6 | 2.Prennerae-1  2.Prennerae-2  6.Prennerae-1  6.Prennerae-2  9.Prennerae-1  9.Prennerae-2 | MirZC047  MirZC047  MirZC103  MirZC103  ZCMV12754  ZCMV12754 | Kirindy  Kirindy  Kirindy  Kirindy  Kirindy  Kirindy |
| Hap_3 | 20 | 10.Prennerae-1  10.Prennerae-2  11.Prennerae-1  11.Prennerae-2  13.Prennerae-2  14.Prennerae-2  15.Prennerae-1  15.Prennerae-2  16.Prennerae-1  16.Prennerae-2  19.Prennerae-1  19.Prennerae-2  21.Prennerae-1  21.Prennerae-2  22.Prennerae-1  22.Prennerae-2  23.Prennerae-1  23.Prennerae-2  24.Prennerae-1  24.Prennerae-2 | ZCMV12789  ZCMV12789  ZSM 0850/2010 (ZCMV12791)  ZSM 0850/2010 (ZCMV12791)  ACZCV 0525  ACZCV 0526  ACZC1950  ACZC1950  ACZC10445  ACZC10445  ACZCV 0745  ACZCV 0745  ACZC10892  ACZC10892  ACZC10899  ACZC10899  ACZC10906  ACZC10906  ACZC10911  ACZC10911 | Anja Reserve  Anja Reserve  Anja Reserve  Anja Reserve  Anja Reserve  Anja Reserve  Anja Reserve  Anja Reserve  Anja Reserve  Anja Reserve  Sakaviro  Sakaviro  Sakaviro  Sakaviro  Sakaviro  Sakaviro  Sakaviro  Sakaviro  Sakaviro  Sakaviro |
| Hap_4 | 1 | 20.Prennerae-2 | ACZCV 0746 | Sakaviro |
| Hap_5 | 9 | 32.PlinD-1  33.PlinD-1  33.PlinD-2  34.PlinD-1  34.PlinD-2  35.PlinD-1  36.PlinD-1  37.PlinD-1  39.PlinD-1 | ZCMV12790  ACZCV 0528  ACZCV 0528  ACZCV 0300  ACZCV 0300  ACZC1930  ACZC1991  ACZC10441  ACZCV 0782 | Anja Reserve  Anja Reserve  Anja Reserve  Anja Reserve  Anja Reserve  Anja Reserve  Anja Reserve  Anja Reserve  Tsaranoro |
| Hap_6 | 7 | 32.PlinD-2  35.PlinD-2  36.PlinD-2  37.PlinD-2  38.PlinD-1  38.PlinD-2  39.PlinD-2 | ZCMV12790  ACZC1930  ACZC1991  ACZC10441  ACZCV 0777  ACZCV 0777  ACZCV 0782 | Anja Reserve  Anja Reserve  Anja Reserve  Anja Reserve  Tsaranoro  Tsaranoro  Tsaranoro |
| Hap_7 | 10 | 40.Pbastardi-1  40.Pbastardi-2  41.Pbastardi-1  41.Pbastardi-2  42.Pbastardi-1  42.Pbastardi-2  43.Pbastardi-1  43.Pbastardi-2  44.Pbastardi-1  44.Pbastardi-2 | ZSM 0178/2004 (FGZC0327)  ZSM 0178/2004 (FGZC0327)  ZSM 0180/2004 (FGZC0332)  ZSM 0180/2004 (FGZC0332)  FGMV2002.B47  FGMV2002.B47  ZSM 0042/2004 (FGZC0070)  ZSM 0042/2004 (FGZC0070)  FGZC0313  FGZC0313 | Tranoroa  Tranoroa  Tranoroa  Tranoroa  Tolagnaro  Tolagnaro  Tolagnaro  Tolagnaro  Tolagnaro  Tolagnaro |
| Hap_8 | 4 | 45.Pguibeae-1  45.Pguibeae-2  46.Pguibeae-1  46.Pguibeae-2 | ACZC1828  ACZC1828  ACZC6464 (FAZC14657)  ACZC6464 (FAZC14657) | Isalo  Isalo  Isalo  Isalo |
| Hap_9 | 4 | 47.Pguibeae-1  47.Pguibeae-2  48.Pguibeae-1  48.Pguibeae-2 | MRSN R2529  MRSN R2529  MRSN R2568  MRSN R2568 | Isalo  Isalo  Isalo  Isalo |
| Hap_10 | 5 | 49.Pguibeae-1  50.Pguibeae-1  50.Pguibeae-2  52.Pguibeae-1  53.Pguibeae-1 | FGMV2002.1523  FGMV2002.1524  FGMV2002.1524  FGMV2002.1577  ZSM 1101/2003 (FGMV2002.1600) | Toliara  Toliara  Toliara  Toliara  Toliara |
| Hap_11 | 3 | 49.Pguibeae-2  52.Pguibeae-2  53.Pguibeae-2 | FGMV2002.1523  FGMV2002.1577  ZSM 1101/2003 (FGMV2002.1600) | Toliara  Toliara  Toliara |
| Hap_12 | 1 | 51.Pguibeae-1 | FGMV2002.1576 | Toliara |
| Hap_13 | 1 | 51.Pguibeae-2 | FGMV2002.1576 | Toliara |
| Hap_14 | 13 | 54.Pguibeae-1  54.Pguibeae-2  55.Pguibeae-1  55.Pguibeae-2  56.Pguibeae-1  56.Pguibeae-2  58.Pibityensis-1  58.Pibityensis-2  59.Pibityensis-1  59.Pibityensis-2  60.Pibityensis-1  61.Pibityensis-1  61.Pibityensis-2 | ZSM 0187/2004 (FGZC0352)  ZSM 0187/2004 (FGZC0352)  ZSM 0188/2004 (FGZC0353)  ZSM 0188/2004 (FGZC0353)  ZSM 0189/2004 (FGZC0354)  ZSM 0189/2004 (FGZC0354)  FGMV2002.0990  FGMV2002.0990  FGMV2002.0991  FGMV2002.0991  FGMV2002.0992  FGMV2002.0993  FGMV2002.0993 | Tranoroa  Tranoroa  Tranoroa  Tranoroa  Tranoroa  Tranoroa  Ibity  Ibity  Ibity  Ibity  Ibity  Ibity  Ibity |
| Hap_15 | 2 | 57.Pibityensis-1  57.Pibityensis-2 | FGMV2001.D37  FGMV2001.D37 | Itremo  Itremo |
| Hap_16 | 1 | 60.Pibityensis-2 | FGMV2002.0992 | Ibity |
| Hap_17 | 1 | 62.Ptanjaka-1 | ZSM 53/2006 (FGZC0780) | Bemaraha |
| Hap_18 | 1 | 62.Ptanjaka-2 | ZSM 53/2006 (FGZC0780) | Bemaraha |
| Hap_19 | 7 | 63.Ptanjaka-1  65.Ptanjaka-1  68.Ptanjaka-1  68.Ptanjaka-2  69.Ptanjaka-1  71.Ptanjaka-1  72.Ptanjaka-1 | FGZC0953  FGZC0968  FGZC0754  FGZC0754  ZSM 43/2006 (FGZC0755)  ZSM 143/2006 (FGZC0951)  FGZC0952 | Bemaraha  Bemaraha  Bemaraha Bemaraha Bemaraha  Bemaraha Bemaraha |
| Hap_20 | 11 | 63.Ptanjaka-2  65.Ptanjaka-2  66.Ptanjaka-1  66.Ptanjaka-2  67.Ptanjaka-1  67.Ptanjaka-2  69.Ptanjaka-2  70.Ptanjaka-1  70.Ptanjaka-2  71.Ptanjaka-2  72.Ptanjaka-2 | FGZC0953  FGZC0968  ZSM 149/2006 (FGZC0969)  ZSM 149/2006 (FGZC0969)  FGZC0753  FGZC0753  ZSM 43/2006 (FGZC0755)  ZSM 142/2006 (FGZC0950)  ZSM 142/2006 (FGZC0950)  ZSM 143/2006 (FGZC0951)  FGZC0952 | Bemaraha  Bemaraha  Bemaraha  Bemaraha Bemaraha Bemaraha  Bemaraha Bemaraha  Bemaraha  Bemaraha Bemaraha |
| Hap_21 | 1 | 64.Ptanjaka-1 | FGZC0967 | Bemaraha |
| Hap_22 | 1 | 64.Ptanjaka-2 | FGZC0967 | Bemaraha |
| Hap_23 | 2 | 73.Pneglecta-1  73.Pneglecta-2 | ZSM 163/2006 (FGZC0991)  ZSM 163/2006 (FGZC0991) | Bemaraha  Bemaraha |
