# Supplementary material for: ﻿Another step through the crux: a new microendemic rock-dwelling Paroedura (Squamata, Gekkonidae) from south-central Madagascar
Source: Zookeys. 2023 Oct 4;1181:125–54. doi: 10.3897/zookeys.1181.108134 (PMC10568478; doi:10.3897/zookeys.1181.108134)
Supplement: Supplementary material 5 — Morphological dataset [file zookeys-1181-125_article-108134__-s005.docx]

Appendix 5. Morphological dataset used in the analysis of the phenotypic variation in the *Paroedura bastardi* clade.

Appendix 5.1. List of specimens included in the morphological analysis (PCA). Specimens molecularly assigned to a lineage are in bold. Ad = adults; Sub = subadults. Measurements expressed in mm. See Methods for the morphological characters explanations.

| **Species** | **Coll. Numb.** | **Stage** | **SnoutS** | **IO** | **SO** | **SVL** | **TL** | **HL** | **HW** | **HH** | **distE** | **AGL** | **ED** | **EO** | **HAL** | **TIBL** |
| --- | --- | --- | --- | --- | --- | --- | --- | --- | --- | --- | --- | --- | --- | --- | --- | --- |
| ***P. manongavato* sp. nov.** | **ACZCV 0300** | Ad | s | 6 | 5/4 | 68.3 | 49.0 | 19.9 | 15.3 | 8.7 | 3.1 | 30.9 | 5.6 | 2.8 | 7.1 | 11.8 |
| ***P. manongavato* sp. nov.** | **ACZC10442/ACP4725** | Ad | s/i | 6 | 4/5 | 73.0 | 39.9 | 24.1 | 19.3 | — | 3.3 | — | 5.4 | — | 7.5 | — |
| ***P. guibeae*** | **ZSM 0189/2004** | Ad | c | 3 | 4/4 | 56.9 | 33.3 | 16.8 | 14.4 | 7.7 | 2.0 | 26.0 | 3.8 | 2.1 | 6.4 | 9.9 |
| ***P. guibeae*** | **ZSM 0187/2004** | Ad | c/i | 4 | 4/4 | 47.9 | 38.2 | 14.7 | 11.2 | 5.0 | 1.7 | 20.7 | 3.4 | 3.3 | 5.7 | 8.8 |
| ***P. guibeae*** | **ZSM 1101/2003** | Ad | s | 5 | 4/4 | 53.1 | 32.9 | 15.9 | 12.6 | 7.1 | 2.4 | 22.7 | 3.6 | 1.4 | 6.5 | 10.3 |
| ***P. guibeae*** | ZSM 0057/2004 | Ad | c/i | 5 | 4/4 | 49.4 | 25.3 | 14.6 | 11.4 | 5.5 | 1.2 | 20.5 | 2.9 | 1.1 | 6.0 | 9.5 |
| ***P. guibeae*** | ZSM 0058/2004 | Ad | c/i | 4 | 4/5 | 54.2 | 33.2 | 16.0 | 13.3 | 6.7 | 2.0 | 20.3 | 3.6 | 1.7 | 6.0 | 9.5 |
| ***P. guibeae*** | ZSM 590/2000 | Ad | c | 4 | 4/4 | 50.2 | 37.6 | 14.5 | 11.7 | 6.3 | 1.7 | 22.4 | 3.9 | 1.6 | 4.6 | 9.8 |
| ***Paroedura rennerae*** | **ZSM 0849/2010** | Ad | c | 5 | 5/5 | 73.6 | — | 21.2 | 17.1 | 10.4 | 2.7 | 32.2 | 5.3 | 2.7 | 8.6 | 13.0 |
| ***Paroedura rennerae*** | **ZSM 0850/2010** | Ad | s/i | 4 | 5/5 | 80.9 | — | 25.9 | 17.3 | 10.3 | 2.8 | 32.7 | 4.8 | 3.3 | 8.9 | 13.3 |
| ***Paroedura rennerae*** | **ZSM 779/2009** | Sub | s/i | 5 | 4/4 | 49.7 | 42.5 | 16.0 | 12.6 | 7.5 | 2.0 | 18.5 | 3.7 | 1.9 | 6.7 | 9.6 |
| ***Paroedura rennerae*** | **ACZCV 0761** | Sub | c/i | 5 | 5/5 | 61.2 | 40.0 | 18.5 | 14.8 | 7.8 | 2.9 | 26.2 | 4.5 | 2.8 | 7.4 | 12.2 |
| ***Paroedura rennerae*** | **ACZCV 0526** | Sub | i | 3 | 5/6 | 60.9 | 44.4 | 18.9 | 15.1 | 7.9 | 2.7 | 23.2 | 5.0 | 2.9 | 7.4 | 11.0 |
| ***Paroedura rennerae*** | **ACZCV 0804** | Ad | s/i | 5 | 4/4 | 75.2 | 35.9 | 22.3 | 18.7 | 9.3 | 3.8 | 33.0 | 6.0 | 3.2 | 8.4 | 13.6 |
| ***Paroedura rennerae*** | **ACZCV 0525** | Ad | c/i | 5 | 5/5 | 79.7 | 37.5 | 22.4 | 19.1 | 18.6 | 3.2 | 32.8 | 5.9 | 3.8 | 8.8 | 14.2 |
| ***P. bastardi*** | **ZSM 0180/2004** | Sub | c | 6 | 3/3 | 57.1 | 47.5 | 18.2 | 13.6 | 8.0 | 2.4 | 25.2 | 4.3 | 2.2 | 7.5 | 11.0 |
| ***P. bastardi*** | ZFMK 53155 | Ad | s | 5 | 4/5 | 67.4 | 44.3 | 20.5 | 16.7 | 10.6 | 2.8 | 29.5 | 5.2 | 2.1 | 6.9 | 12.1 |
| ***P. bastardi*** | ZFMK 48433 | Ad | s | 4 | 4/4 | 75.1 | 34.1 | 22.7 | 18.2 | 8.6 | 2.9 | 34 | 5.1 | 3.7 | 7.9 | 12.2 |
| ***P. bastardi*** | ZFMK 52300 | Ad | c/s | 4 | 3/4 | 61.1 | 47.8 | 19.1 | 15.5 | 8.2 | 2.4 | 27.2 | 4.0 | 3.1 | 7.6 | 10.7 |
| ***P. bastardi*** | ZFMK 20728 | Sub | s | 5 | 4/4 | 54.4 | 33.7 | 17.7 | 14.1 | 8.6 | 2.5 | 22.5 | 4.1 | 1.7 | 6.5 | 10.1 |
| ***P. bastardi*** | ZSM 0202/2002 | Ad | s | 6 | 4/4 | 78.5 | 53.6 | 24.1 | 20.9 | 10.6 | 3.4 | 33.9 | 5.3 | 2.2 | 9.0 | 13.5 |
| ***P. bastardi*** | MNHN 1900.0006 | Ad | s | 5 | 4/4 | 64.6 | 39.5 | 21.7 | 17.6 | 10.0 | 3.2 | 26.1 | 4.6 | 2.4 | 8.0 | 12.4 |
| ***P. bastardi*** | MNHN 1899.0337 | Ad | s | 5 | 3/3 | 58.8 | — | 18.6 | 14.3 | 8.5 | 2.4 | 22.5 | 4.1 | 2.2 | 6.9 | 10.6 |
| ***P. ibityensis*** | ZSM 483/2001 | Ad | s | 4 | 4/4 | 58.2 | — | 15.3 | 12.9 | 7.1 | 2.2 | 22.5 | 3.7 | 1.7 | 6.4 | 9.3 |
| ***P. ibityensis*** | ZSM 482/2001 | Ad | s | 5 | 3/3 | 62.2 | 39.8 | 16.5 | 14.4 | 8.4 | 2.2 | 24.8 | 3.7 | 2.0 | 6.9 | 10.9 |
| ***P. ibityensis*** | ZSM 88/2009 | Ad | — | — | — | 56.9 | 42.7 | 18.1 | 14.7 | 7.4 | 2.6 | 23.1 | 3.9 | 2.8 | 7.2 | 9.9 |
| ***P. ibityensis*** | ZSM 89/2009 | Ad | — | — | — | 62.1 | 55.7 | 15.7 | 13.3 | 6.7 | 2.5 | 25.5 | 4 | 2.2 | 6.2 | 9.5 |
| ***P. ibityensis*** | ZSM 85/2009 | Ad | — | — | — | 55.1 | — | 16.3 | 14 | 6.5 | 2.3 | 25 | 4.1 | 2 | 6.9 | 10.1 |
| ***P. ibityensis*** | ZSM 86/2009 | Ad | — | — | — | 54.3 | 20.9 | 17.0 | 13.8 | 6.8 | 2.7 | 26.6 | 3.7 | 1.5 | 6.4 | 9.2 |

Appendix 5.2. Variable contributions to the first five axes (PCs) of the Principal Components Analysis (PCA) on the morphological (quantitative and qualitative) dataset. In bold contributions > 0.70. Variables marked with /SVL were divided by SVL.

|  | PC.1 | PC.2 | PC.3 | PC.4 | PC.5 |
| --- | --- | --- | --- | --- | --- |
| Quantitative |  |  |  |  |  |
| IO | 0.4058 | 0.1797 | -0.5238 | -0.0600 | 0.5302 |
| SO | -0.0078 | 0.5309 | 0.5243 | -0.2523 | -0.0913 |
| SVL | -0.0357 | **0.8474** | -0.0179 | -0.1250 | -0.0025 |
| TL/SVL | 0.0326 | -0.5778 | 0.3734 | 0.0225 | 0.5425 |
| HL/SVL | **0.7592** | -0.0732 | 0.0434 | 0.0018 | -0.3733 |
| HW/SVL | **0.7680** | 0.0208 | -0.1276 | 0.0611 | -0.3409 |
| HH/SVL | 0.3977 | 0.4315 | -0.0853 | -0.5700 | 0.1064 |
| distE/SVL | 0.6602 | 0.3129 | 0.0092 | 0.2531 | 0.1153 |
| AGL/SVL | 0.0972 | 0.2731 | -0.1986 | **0.7954** | -0.0584 |
| ED/SVL | 0.5974 | 0.2332 | 0.3832 | 0.1931 | 0.4443 |
| EO/SVL | 0.1527 | 0.0443 | **0.7540** | 0.1645 | -0.0926 |
| HAL/SVL | 0.5321 | -0.5394 | 0.0044 | -0.1808 | -0.2604 |
| TIBL/SVL | 0.6043 | -0.4037 | -0.0503 | -0.1541 | 0.1424 |
| Quantitative |  |  |  |  |  |
| SnoutS | 0.0389 | 0.0794 | 0.4063 | 0.1848 | 0.0765 |
| percentage of variance | 23.6245 | 17.8529 | 11.7267 | 9.7955 | 9.2525 |
